# Supplementary material for: Four-band non-Abelian topological insulator and its experimental realization
Source: Nat Commun. 2021 Nov 9;12:6471. doi: 10.1038/s41467-021-26763-1 (PMC8578628; doi:10.1038/s41467-021-26763-1)
Supplement: Supplementary file 1 — Supplementary Information [file 41467_2021_26763_MOESM1_ESM.pdf]

Supplementary Information: Four-band non-Abelian topological insulator and its experimental realization

Tianshu Jiang[1†], Qinghua Guo[1†], Ruo-Yang Zhang[1], Zhao-Qing Zhang[1], Biao Yang[1,2*], C. T. Chan[1*]

[1]Department of Physics and Institute for Advanced Study, The Hong Kong University of Science and Technology, Hong Kong, China
[2]College of Advanced Interdisciplinary Studies, National University of Defense Technology, Changsha 410073, China

[†]These authors contributed equally to this work.
[*]Correspondence to: yangbiaocam@nudt.edu.cn; phchan@ust.hk;

Contents

Supplementary Note 1. Non-Abelian topological charges in four-band models 2

Supplementary Note 2. Rotations in four-dimension 4

Supplementary Note 3. Tight-binding model 6

Supplementary Note 4. Analytical solutions of edge states for the flat-band models 9

(i) Edge states of the charges $\pm q_{mn}$ 9

(ii) Edge states of the charges $\pm q_{1234}$ 10

(iii) Evolution of edge states of the charges $\pm q_{1234}$ between different factorizations 10

(iv) Edge states of the charge -1 represented by q_{mn}^2 11

(v) Evolution of edge states of the charge -1 12

Reference: 15

Supplementary Figures 16

Supplementary Note 1. Non-Abelian topological charges in four-band models

The calculation[1] of non-Abelian topological charges requires lifting the Berry connection one-form from the Lie algebra $\mathfrak{so}(4)$ to $\mathfrak{spin}(4)$, i.e.,

$$L_{ij} \to t_{ij} = -\frac{1}{4}\left[\Gamma_i, \Gamma_j\right] \in \mathfrak{spin}(4) \tag{1}$$

where the corresponding coefficients β_a^{ij} are kept as,

$$[A(k)]_a = \sum_{i<j} \beta_a^{ij}(k) L_{ij} \overset{lift}{\longrightarrow} [\bar{A}(k)]_a = \sum_{i<j} \beta_a^{ij}(k) t_{ij} \tag{2}$$

We set the basis of the Lie algebra $\mathfrak{so}(4)$ as $\left(L_{ij}\right)_{a,b=1:4} = -\delta_{ia}\delta_{jb} + \delta_{ib}\delta_{ja}$, the explicit forms are,

$$\mathrm{L}_{12} = \begin{pmatrix} 0 & -1 & 0 & 0 \\ 1 & 0 & 0 & 0 \\ 0 & 0 & 0 & 0 \\ 0 & 0 & 0 & 0 \end{pmatrix}, \mathrm{L}_{13} = \begin{pmatrix} 0 & 0 & -1 & 0 \\ 0 & 0 & 0 & 0 \\ 1 & 0 & 0 & 0 \\ 0 & 0 & 0 & 0 \end{pmatrix}, \mathrm{L}_{14} = \begin{pmatrix} 0 & 0 & 0 & -1 \\ 0 & 0 & 0 & 0 \\ 0 & 0 & 0 & 0 \\ 1 & 0 & 0 & 0 \end{pmatrix};$$
$$\mathrm{L}_{23} = \begin{pmatrix} 0 & 0 & 0 & 0 \\ 0 & 0 & -1 & 0 \\ 0 & 1 & 0 & 0 \\ 0 & 0 & 0 & 0 \end{pmatrix}, \mathrm{L}_{24} = \begin{pmatrix} 0 & 0 & 0 & 0 \\ 0 & 0 & 0 & -1 \\ 0 & 0 & 0 & 0 \\ 0 & 1 & 0 & 0 \end{pmatrix}, \mathrm{L}_{34} = \begin{pmatrix} 0 & 0 & 0 & 0 \\ 0 & 0 & 0 & 0 \\ 0 & 0 & 0 & -1 \\ 0 & 0 & 1 & 0 \end{pmatrix} \tag{3}$$

We use the following Γ matrices,

$$\Gamma_1 = \begin{pmatrix} 0 & 0 & 0 & i \\ 0 & 0 & i & 0 \\ 0 & -i & 0 & 0 \\ -i & 0 & 0 & 0 \end{pmatrix}, \Gamma_2 = \begin{pmatrix} 0 & 0 & 0 & 1 \\ 0 & 0 & -1 & 0 \\ 0 & -1 & 0 & 0 \\ 1 & 0 & 0 & 0 \end{pmatrix}$$
$$\Gamma_3 = \begin{pmatrix} 0 & 0 & i & 0 \\ 0 & 0 & 0 & -i \\ -i & 0 & 0 & 0 \\ 0 & i & 0 & 0 \end{pmatrix}, \Gamma_4 = \begin{pmatrix} 0 & 0 & 1 & 0 \\ 0 & 0 & 0 & 1 \\ 1 & 0 & 0 & 0 \\ 0 & 1 & 0 & 0 \end{pmatrix} \tag{4}$$

They are obtained from,

$$\Gamma_1 = -\sigma_2 \otimes \sigma_1, \Gamma_2 = -\sigma_2 \otimes \sigma_2, \Gamma_3 = -\sigma_2 \otimes \sigma_3, \Gamma_4 = \sigma_1 \otimes \sigma_0 \tag{5}$$

where $\sigma_i (i = 1,2,3)$ are Pauli matrices and σ_0 is the 2×2 identity matrix. They satisfy the anticommutation relations $\left\{\Gamma_i, \Gamma_j\right\} = 2\delta_{ij}$.

The basis of Clifford algebra $C\ell_{0,3}$ that generates the group Q_{16} is obtained by,

$$e_{j-1} \equiv \exp\left(-\frac{\pi}{4}\left[\Gamma_1, \Gamma_j\right]\right) = \frac{1}{2}\left[\Gamma_j, \Gamma_1\right] \tag{6}$$

for $2 \le j \le 4$. Their explicit forms are,

$$e_1 = \frac{1}{2}[\Gamma_2, \Gamma_1] = \begin{pmatrix} -i & 0 & 0 & 0 \\ 0 & i & 0 & 0 \\ 0 & 0 & -i & 0 \\ 0 & 0 & 0 & i \end{pmatrix}$$

$$e_2 = \frac{1}{2}[\Gamma_3, \Gamma_1] = \begin{pmatrix} 0 & 1 & 0 & 0 \\ -1 & 0 & 0 & 0 \\ 0 & 0 & 0 & 1 \\ 0 & 0 & -1 & 0 \end{pmatrix}$$

$$e_3 = \frac{1}{2}[\Gamma_4, \Gamma_1] = \begin{pmatrix} 0 & -i & 0 & 0 \\ -i & 0 & 0 & 0 \\ 0 & 0 & 0 & i \\ 0 & 0 & i & 0 \end{pmatrix} \quad (7)$$

with $e_i^2 = -I_{4\times 4}$ and $e_i e_j = -e_j e_i$ (or we combine them two together as $\{e_i, e_j\} = -2\delta_{ij}$).

From the basis $\{e_1, e_2, e_3\}$, we have,

$$Q_{16} = \bigcup_{n_i \in \{0,1\}} \{\pm e_1^{n_1} e_2^{n_2} e_3^{n_3}\}$$

$$= \{\pm e_1, \pm e_2, \pm e_3, \pm e_1 e_2, \pm e_1 e_3, \pm e_2 e_3\} \cup \{+1, -1, +e_1 e_2 e_3, -e_1 e_2 e_3\} \quad (8)$$

where,

$$e_{12} \equiv e_1 e_2 = \begin{pmatrix} 0 & -i & 0 & 0 \\ -i & 0 & 0 & 0 \\ 0 & 0 & 0 & -i \\ 0 & 0 & -i & 0 \end{pmatrix}, e_{13} \equiv e_1 e_3 = \begin{pmatrix} 0 & -1 & 0 & 0 \\ 1 & 0 & 0 & 0 \\ 0 & 0 & 0 & 1 \\ 0 & 0 & -1 & 0 \end{pmatrix}$$

$$e_{23} \equiv e_2 e_3 = \begin{pmatrix} -i & 0 & 0 & 0 \\ 0 & i & 0 & 0 \\ 0 & 0 & i & 0 \\ 0 & 0 & 0 & -i \end{pmatrix}, e_{123} \equiv e_1 e_2 e_3 = \begin{pmatrix} -1 & 0 & 0 & 0 \\ 0 & -1 & 0 & 0 \\ 0 & 0 & 1 & 0 \\ 0 & 0 & 0 & 1 \end{pmatrix} \quad (9)$$

We rename each Q_{16} group element as (to be more convenient in physics analysis; also see Table 1 in the main text),

$$e_1 \to q_{12}, e_2 \to q_{13}, e_3 \to q_{14}, e_{12} \to q_{23}, e_{13} \to q_{24}, e_{23} \to q_{34}, e_{123} \to q_{1234} \quad (10)$$

Finally, we have the multiplication tables as shown in Supplementary Table 1 and 2.

Supplementary Table 1. Multiplication table of Q_{16} group labelled with Clifford algebra basis.

Q_{16} multiplication table

	+1	e_1	e_2	e_3	e_{12}	e_{13}	e_{23}	e_{123}	-1	$-e_1$	$-e_2$	$-e_3$	$-e_{12}$	$-e_{13}$	$-e_{23}$	$-e_{123}$
+1	+1	e_1	e_2	e_3	e_{12}	e_{13}	e_{23}	e_{123}	-1	$-e_1$	$-e_2$	$-e_3$	$-e_{12}$	$-e_{13}$	$-e_{23}$	$-e_{123}$
e_1	e_1	-1	e_{12}	e_{13}	$-e_2$	$-e_3$	e_{123}	$-e_{23}$	$-e_1$	+1	$-e_{12}$	$-e_{13}$	e_2	e_3	$-e_{123}$	e_{23}
e_2	e_2	$-e_{12}$	-1	e_{23}	e_1	$-e_{123}$	$-e_3$	e_{13}	$-e_2$	e_{12}	+1	$-e_{23}$	$-e_1$	e_{123}	e_3	$-e_{13}$
e_3	e_3	$-e_{13}$	$-e_{23}$	-1	e_{123}	e_1	e_2	$-e_{12}$	$-e_3$	e_{13}	e_{23}	+1	$-e_{123}$	$-e_1$	$-e_2$	e_{12}
e_{12}	e_{12}	e_2	$-e_1$	e_{123}	-1	e_{23}	$-e_{13}$	$-e_3$	$-e_{12}$	$-e_2$	e_1	$-e_{123}$	+1	$-e_{23}$	e_{13}	e_3
e_{13}	e_{13}	e_3	$-e_{123}$	$-e_1$	$-e_{23}$	-1	e_{12}	e_2	$-e_{13}$	$-e_3$	e_{123}	e_1	e_{23}	+1	$-e_{12}$	$-e_2$
e_{23}	e_{23}	e_{123}	e_3	$-e_2$	e_{13}	$-e_{12}$	-1	$-e_1$	$-e_{23}$	$-e_{123}$	$-e_3$	e_2	$-e_{13}$	e_{12}	+1	e_1
e_{123}	e_{123}	$-e_{23}$	e_{13}	$-e_{12}$	$-e_3$	e_2	$-e_1$	+1	$-e_{123}$	e_{23}	$-e_{13}$	e_{12}	e_3	$-e_2$	e_1	-1
-1	-1	$-e_1$	$-e_2$	$-e_3$	$-e_{12}$	$-e_{13}$	$-e_{23}$	$-e_{123}$	+1	e_1	e_2	e_3	e_{12}	e_{13}	e_{23}	e_{123}
$-e_1$	$-e_1$	+1	$-e_{12}$	$-e_{13}$	e_2	e_3	$-e_{123}$	e_{23}	e_1	-1	e_{12}	e_{13}	$-e_2$	$-e_3$	e_{123}	$-e_{23}$
$-e_2$	$-e_2$	e_{12}	+1	$-e_{23}$	$-e_1$	e_{123}	e_3	$-e_{13}$	e_2	$-e_{12}$	-1	e_{23}	e_1	$-e_{123}$	$-e_3$	e_{13}
$-e_3$	$-e_3$	e_{13}	e_{23}	+1	$-e_{123}$	$-e_1$	$-e_2$	e_{12}	e_3	$-e_{13}$	$-e_{23}$	-1	e_{123}	e_1	e_2	$-e_{12}$
$-e_{12}$	$-e_{12}$	$-e_2$	e_1	$-e_{123}$	+1	$-e_{23}$	e_{13}	e_3	e_{12}	e_2	$-e_1$	e_{123}	-1	e_{23}	$-e_{13}$	$-e_3$
$-e_{13}$	$-e_{13}$	$-e_3$	e_{123}	e_1	e_{23}	+1	$-e_{12}$	$-e_2$	e_{13}	e_3	$-e_{123}$	$-e_1$	$-e_{23}$	-1	e_{12}	e_2
$-e_{23}$	$-e_{23}$	$-e_{123}$	$-e_3$	e_2	$-e_{13}$	e_{12}	+1	e_1	e_{23}	e_{123}	e_3	$-e_2$	e_{13}	$-e_{12}$	-1	$-e_1$
$-e_{123}$	$-e_{123}$	e_{23}	$-e_{13}$	e_{12}	e_3	$-e_2$	e_1	-1	e_{123}	$-e_{23}$	e_{13}	$-e_{12}$	$-e_3$	e_2	$-e_1$	+1

Supplementary Table 2. Multiplication table of Q_{16} group labelled with band-index.

Q_{16} multiplication table

	+1	q_{12}	q_{13}	q_{14}	q_{23}	q_{24}	q_{34}	q_{1234}	-1	$-q_{12}$	$-q_{13}$	$-q_{14}$	$-q_{23}$	$-q_{24}$	$-q_{34}$	$-q_{1234}$
+1	+1	q_{12}	q_{13}	q_{14}	q_{23}	q_{24}	q_{34}	q_{1234}	-1	$-q_{12}$	$-q_{13}$	$-q_{14}$	$-q_{23}$	$-q_{24}$	$-q_{34}$	$-q_{1234}$
q_{12}	q_{12}	-1	q_{23}	q_{24}	$-q_{13}$	$-q_{14}$	q_{1234}	$-q_{34}$	$-q_{12}$	+1	$-q_{23}$	$-q_{24}$	q_{13}	q_{14}	$-q_{1234}$	q_{34}
q_{13}	q_{13}	$-q_{23}$	-1	q_{34}	q_{12}	$-q_{1234}$	$-q_{14}$	q_{24}	$-q_{13}$	q_{23}	+1	$-q_{34}$	$-q_{12}$	q_{1234}	q_{14}	$-q_{24}$
q_{14}	q_{14}	$-q_{24}$	$-q_{34}$	-1	q_{1234}	q_{12}	q_{13}	$-q_{23}$	$-q_{14}$	q_{24}	q_{34}	+1	$-q_{1234}$	$-q_{12}$	$-q_{13}$	q_{23}
q_{23}	q_{23}	q_{13}	$-q_{12}$	q_{1234}	-1	q_{34}	$-q_{24}$	$-q_{14}$	$-q_{23}$	$-q_{13}$	q_{12}	$-q_{1234}$	+1	$-q_{34}$	q_{24}	q_{14}
q_{24}	q_{24}	q_{14}	$-q_{1234}$	$-q_{12}$	$-q_{34}$	-1	q_{23}	q_{13}	$-q_{24}$	$-q_{14}$	q_{1234}	q_{12}	q_{34}	+1	$-q_{23}$	$-q_{13}$
q_{34}	q_{34}	q_{1234}	q_{14}	$-q_{13}$	q_{24}	$-q_{23}$	-1	$-q_{12}$	$-q_{34}$	$-q_{1234}$	$-q_{14}$	q_{13}	$-q_{24}$	q_{23}	+1	q_{12}
q_{1234}	q_{1234}	$-q_{34}$	q_{24}	$-q_{23}$	$-q_{14}$	q_{13}	$-q_{12}$	+1	$-q_{1234}$	q_{34}	$-q_{24}$	q_{23}	q_{14}	$-q_{13}$	q_{12}	-1
-1	-1	$-q_{12}$	$-q_{13}$	$-q_{14}$	$-q_{23}$	$-q_{24}$	$-q_{34}$	$-q_{1234}$	+1	q_{12}	q_{13}	q_{14}	q_{23}	q_{24}	q_{34}	q_{1234}
$-q_{12}$	$-q_{12}$	+1	$-q_{23}$	$-q_{24}$	q_{13}	q_{14}	$-q_{1234}$	q_{34}	q_{12}	-1	q_{23}	q_{24}	$-q_{13}$	$-q_{14}$	q_{1234}	$-q_{34}$
$-q_{13}$	$-q_{13}$	q_{23}	+1	$-q_{34}$	$-q_{12}$	q_{1234}	q_{14}	$-q_{24}$	q_{13}	$-q_{23}$	-1	q_{34}	q_{12}	$-q_{1234}$	$-q_{14}$	q_{24}
$-q_{14}$	$-q_{14}$	q_{24}	q_{34}	+1	$-q_{1234}$	$-q_{12}$	$-q_{13}$	q_{23}	q_{14}	$-q_{24}$	$-q_{34}$	-1	q_{1234}	q_{12}	q_{13}	$-q_{23}$
$-q_{23}$	$-q_{23}$	$-q_{13}$	q_{12}	$-q_{1234}$	+1	$-q_{34}$	q_{24}	q_{14}	q_{23}	q_{13}	$-q_{12}$	q_{1234}	-1	q_{34}	$-q_{24}$	$-q_{14}$
$-q_{24}$	$-q_{24}$	$-q_{14}$	q_{1234}	q_{12}	q_{34}	+1	$-q_{23}$	$-q_{13}$	q_{24}	q_{14}	$-q_{1234}$	$-q_{12}$	$-q_{34}$	-1	q_{23}	q_{13}
$-q_{34}$	$-q_{34}$	$-q_{1234}$	$-q_{14}$	q_{13}	$-q_{24}$	q_{23}	+1	q_{12}	q_{34}	q_{1234}	q_{14}	$-q_{13}$	q_{24}	$-q_{23}$	-1	$-q_{12}$
$-q_{1234}$	$-q_{1234}$	q_{34}	$-q_{24}$	q_{23}	q_{14}	$-q_{13}$	q_{12}	-1	q_{1234}	$-q_{34}$	q_{24}	$-q_{23}$	$-q_{14}$	q_{13}	$-q_{12}$	+1

Supplementary Note 2. Rotations in four-dimension

We briefly recall some facts about rotations in the four-dimension[2-4]. For each rotation R, there is at least one pair of orthogonal 2-planes (the 2-planes are also dubbed as invariant planes) - A and B which are invariant under the rotation R and span the four-dimensional space, i.e. for any $\vec{a} \in A$ and $\vec{b} \in B$ we have $\vec{a} \perp \vec{b}$, $R\vec{a} \in A$ and $R\vec{b} \in B$. We define the angle between $\vec{a}$ and

$R\vec{a}$ ($\vec{b}$ and $R\vec{b}$) in the 2-plane A (B) as α (β). As thus, four-dimensional rotations can be categorized into two types: simple rotations ($\alpha = 0$ and $\beta \neq 0$ or $\alpha \neq 0$ and $\beta = 0$) and double rotations ($\alpha \neq 0$ and $\beta \neq 0$). When the two rotation angles satisfy $|\alpha| = |\beta|$, the rotation R is called isoclinic rotation, where there are infinitely many pairs of orthogonal 2-planes. Assuming that the coordinate set is ordered as $ouxyz$ with o indicating the origin, we consider two 2-planes $A = oux$ and $B = oyz$ and set the rotation angle α (β) positive from ou to ox (oy to oz). Then isoclinic rotations with $\alpha\beta > 0$ are denoted as left-isoclinic; those with $\alpha\beta < 0$ as right-isoclinic. Note that the two cases with $|\alpha| = |\beta| = 0\ or\ \pi$ are the only ones that are simultaneously left- and right-isoclinic. When the Bloch wave-vector runs across the 1D first Brillouin zone ($k = -\pi \rightarrow \pi$), the rotation matrix $R(k)$ at each k for each charge can be classified to be single or double rotations. The charges $\pm q_{1234}$ have to be realized via double rotations. In some ideal cases, $+/-\ q_{1234}$ consists of purely left/right-isoclinic rotations, where there are infinitely many pairs of orthogonal 2-planes. All the other charges can be ideally described by simple rotations.

It is well known that rotations can be encoded by quaternion ($\mathbb{H}$) multiplication (discovered by Hamilton in 1843 and frequently used in engineering applications). For example, in three-dimension the rotation $\vec{r}'_{3D} = R_{3D}\vec{r}_{3D}$ can be calculated with $r'_{3D} = q r_{3D} q^{-1}$, where $q \in SU(2)$ being the unit quaternion and $\vec{r}_{3D}$ & $\vec{r}'_{3D} \in \mathbb{R}^3$ can be identified with the pure quaternion (no real part), i.e. r_{3D} & $r'_{3D} \in Im(\mathbb{H})$, respectively. Actually the calculation is made by a surjective homomorphism, $\rho: q \in SU(2) \rightarrow R_{3D} \in SO(3)$, whose kernel is $\{1, -1\}$ indicting $SU(2)$ is a double cover of $SO(3)$. In four-dimension, we have similar forms like $r' = q_L r q_R$ where $q_{L,R} \in SU(2)$ and r & $r' \in \mathbb{H}$ are identified with $\vec{r}$ & $\vec{r}' \in \mathbb{R}^4$, respectively. Likewise, a surjective homomorphism $\rho: (q_L, q_R) \in SU(2) \times SU(2) \rightarrow R \in SO(4)$ with kernel being $\{(1,1), (-1,-1)\}$ also enables a double-cover. All above properties can be summarized as $Spin(N)$ is a double cover of $SO(N)$, and there are group isomorphisms $Spin(3) \cong SU(2)$ and $Spin(4) \cong SU(2) \times SU(2)$.

Left/right isoclinic rotations are represented by left/right multiplication of unit quaternions. Thus, any rotation in four-dimension can be factorized into the commutative composition of two isoclinic rotations, i.e. $R = R_{q_L} R_{q_R}$. We denote $R_{q_L}(r) = q_L r$ with $q_L = a + bi + cj + dk$ and $r = u + xi + yj + zk$. Then,

$$R_{q_L}(r) = \begin{pmatrix} a & -b & -c & -d \\ b & a & -d & c \\ c & d & a & -b \\ d & -c & b & a \end{pmatrix} \begin{pmatrix} u \\ x \\ y \\ z \end{pmatrix} \tag{11}$$

This is a left quaternion multiplication of r by q_L. For a right quaternion multiplication, i.e. $R_{q_R}(r) = rq_R$. Assuming $q_R = p + qi + rj + sk$, we have,

$$R_{q_R}(r) = \begin{pmatrix} p & -q & -r & -s \\ q & p & s & -r \\ r & -s & p & q \\ s & r & -q & p \end{pmatrix} \begin{pmatrix} u \\ x \\ y \\ z \end{pmatrix} \tag{12}$$

It is easy to see $R_{q_L}\big(R_{q_R}(r)\big) = R_{q_R}\big(R_{q_L}(r)\big) = q_L r q_R$ where quaternion multiplication is associative. Thus, the two isoclinic rotations are commutative.

Supplementary Note 3. Tight-binding model

The real-space Hamiltonian reads,

$$\mathcal{H} = \sum_n \begin{pmatrix} \sum_{\substack{X=A,B,C,D \\ Y=A,B,C,D}} s_{XY} c_{X,n}^{\dagger} c_{Y,n} + \\ \sum_{\substack{X=A,B,C,D \\ Y=A,B,C,D}} v_{XY} c_{X,n}^{\dagger} c_{Y,n+1} + \sum_{\substack{X=A,B,C,D \\ Y=A,B,C,D}} v_{XYl} c_{X,n}^{\dagger} c_{Y,n+2} + h.c. \end{pmatrix} \tag{13}$$

where $c_{X,n}^{\dagger}$ and $c_{X,n}$ are creation and annihilation operators on the sub-lattice 'X/Y' and site 'n', respectively. Here, we consider a more general case having both the NN (nearest neighbour) and NNN (next-nearest neighbour) hoppings. After Fourier transformation we obtain,

$$H(k) = \begin{bmatrix} s_{AA} + 2v_{AA}\cos k & 2w_{AB}\sin k & 2w_{AC}\sin k & 2w_{AD}\sin k \\ 2w_{AB}\sin k & s_{BB} + 2v_{BB}\cos k & 2w_{BC}\sin k & 2w_{BD}\sin k \\ 2w_{AC}\sin k & 2w_{BC}\sin k & s_{CC} + 2v_{CC}\cos k & 2w_{CD}\sin k \\ 2w_{AD}\sin k & 2w_{BD}\sin k & 2w_{CD}\sin k & s_{DD} + 2v_{DD}\cos k \end{bmatrix}$$

$$+ \begin{bmatrix} 2v_{AAl}\cos 2k & 2w_{ABl}\sin 2k & 2w_{ACl}\sin 2k & 2w_{ADl}\sin 2k \\ 2w_{ABl}\sin 2k & 2v_{BBl}\cos 2k & 2w_{BCl}\sin 2k & 2w_{BDl}\sin 2k \\ 2w_{ACl}\sin 2k & 2w_{BCl}\sin 2k & 2v_{CCl}\cos 2k & 2w_{CDl}\sin 2k \\ 2w_{ADl}\sin 2k & 2w_{BDl}\sin 2k & 2w_{CDl}\sin 2k & 2v_{DDl}\cos 2k \end{bmatrix}$$

$$+ \begin{bmatrix} 0 & s_{AB} + 2r_{AB}\cos k & s_{AC} + 2r_{AC}\cos k & s_{AD} + 2r_{AD}\cos k \\ s_{AB} + 2r_{AB}\cos k & 0 & s_{BC} + 2r_{BC}\cos k & s_{BD} + 2r_{BD}\cos k \\ s_{AC} + 2r_{AC}\cos k & s_{BC} + 2r_{BC}\cos k & 0 & s_{CD} + 2r_{CD}\cos k \\ s_{AD} + 2r_{AD}\cos k & s_{BD} + 2r_{BD}\cos k & s_{CD} + 2r_{CD}\cos k & 0 \end{bmatrix}$$

$$
+\begin{bmatrix} 0 & 2r_{ABl}\cos 2k & 2r_{ACl}\cos 2k & 2r_{ADl}\cos 2k \\ 2r_{ABl}\cos 2k & 0 & 2r_{BCl}\cos 2k & 2r_{BDl}\cos 2k \\ 2r_{ACl}\cos 2k & 2r_{BCl}\cos 2k & 0 & 2r_{CDl}\cos 2k \\ 2r_{ADl}\cos 2k & 2r_{BDl}\cos 2k & 2r_{CDl}\cos 2k & 0 \end{bmatrix} \tag{14}
$$

where we have set,

$$
\begin{gathered}
v_{AB(l)} = r_{AB(l)} + iw_{AB(l)} = v_{BA(l)} \\
v_{AC(l)} = r_{AC(l)} + iw_{AC(l)} = v_{CA(l)} \\
v_{AD(l)} = r_{AD(l)} + iw_{AD(l)} = v_{DA(l)} \\
v_{BC(l)} = r_{BC(l)} + iw_{BC(l)} = v_{CB(l)} \\
v_{BD(l)} = r_{BD(l)} + iw_{BD(l)} = v_{DB(l)} \\
v_{CD(l)} = r_{CD(l)} + iw_{CD(l)} = v_{DC(l)}
\end{gathered} \tag{15}
$$

Supplementary Table 3. Tight-binding coefficients of the ideal flat band models for different non-Abelian topological charges (charge -1 needs next nearing neighbour hoppings).

s_{XX} coefficient

	q_{12}	$-q_{12}$	q_{13}	$-q_{13}$	q_{14}	$-q_{14}$	q_{23}	$-q_{23}$	q_{24}	$-q_{24}$	q_{34}	$-q_{34}$	q_{1234}	$-q_{1234}$	+1	-1
s_{AA}	3/2	3/2	2	2	5/2	5/2	1	1	1	1	1	1	3/2	3/2	1	
s_{BB}	3/2	3/2	2	2	2	2	5/2	5/2	3	3	2	2	3/2	3/2	2	
s_{CC}	3	3	2	2	3	3	5/2	5/2	3	3	7/2	7/2	7/2	7/2	3	
s_{DD}	4	4	4	4	5/2	5/2	4	4	3	3	7/2	7/2	7/2	7/2	4	

v_{XX} coefficient

	q_{12}	$-q_{12}$	q_{13}	$-q_{13}$	q_{14}	$-q_{14}$	q_{23}	$-q_{23}$	q_{24}	$-q_{24}$	q_{34}	$-q_{34}$	q_{1234}	$-q_{1234}$	+1	-1
v_{AA}	1/4	1/4	1/2	1/2	3/4	3/4	0	0	0	0	0	0	1/4	1/4	0	
v_{BB}	-1/4	-1/4	0	0	0	0	1/4	1/4	1/2	1/2	0	0	-1/4	-1/4	0	
v_{CC}	0	0	-1/2	-1/2	0	0	-1/4	-1/4	0	0	1/4	1/4	1/4	1/4	0	
v_{DD}	0	0	0	0	-3/4	-3/4	0	0	-1/2	-1/2	-1/4	-1/4	-1/4	-1/4	0	

w_{XY} coefficient

	q_{12}	$-q_{12}$	q_{13}	$-q_{13}$	q_{14}	$-q_{14}$	q_{23}	$-q_{23}$	q_{24}	$-q_{24}$	q_{34}	$-q_{34}$	q_{1234}	$-q_{1234}$	+1	-1
w_{AB}	1/4	-1/4	0	0	0	0	0	0	0	0	0	0	1/4	-1/4	0	
w_{AC}	0	0	1/2	-1/2	0	0	0	0	0	0	0	0	0	0	0	
w_{AD}	0	0	0	0	3/4	-3/4	0	0	0	0	0	0	0	0	0	
w_{BC}	0	0	0	0	0	0	1/4	-1/4	0	0	0	0	0	0	0	
w_{BD}	0	0	0	0	0	0	0	0	1/2	-1/2	0	0	0	0	0	
w_{CD}	0	0	0	0	0	0	0	0	0	0	1/4	-1/4	1/4	1/4	0	

Supplementary Table 4. Tight-binding coefficients of the ideal flat band models for different factorizations of charges $\pm q_{1234}$.

s_{XX} coefficient

	$q_{1234}=q_{12}q_{34}$	$-q_{1234}=-q_{12}q_{34}$	$q_{1234}=-q_{13}q_{24}$	$-q_{1234}=q_{13}q_{24}$	$q_{1234}=q_{14}q_{23}$	$-q_{1234}=-q_{14}q_{23}$
s_{AA}	3/2	3/2	2	2	5/2	5/2
s_{BB}	3/2	3/2	3	3	5/2	5/2
s_{CC}	7/2	7/2	2	2	5/2	5/2
s_{DD}	7/2	7/2	3	3	5/2	5/2

v_{XX} coefficient

	$q_{1234}=q_{12}q_{34}$	$-q_{1234}=-q_{12}q_{34}$	$q_{1234}=-q_{13}q_{24}$	$-q_{1234}=q_{13}q_{24}$	$q_{1234}=q_{14}q_{23}$	$-q_{1234}=-q_{14}q_{23}$
v_{AA}	1/4	1/4	1/2	1/2	3/4	3/4
v_{BB}	-1/4	-1/4	1/2	1/2	1/4	1/4
v_{CC}	1/4	1/4	-1/2	-1/2	-1/4	-1/4
v_{DD}	-1/4	-1/4	-1/2	-1/2	-3/4	-3/4

w_{XY} coefficient

	$q_{1234}=q_{12}q_{34}$	$-q_{1234}=-q_{12}q_{34}$	$q_{1234}=-q_{13}q_{24}$	$-q_{1234}=q_{13}q_{24}$	$q_{1234}=q_{14}q_{23}$	$-q_{1234}=-q_{14}q_{23}$
w_{AB}	1/4	-1/4	0	0	0	0
w_{AC}	0	0	-1/2	1/2	0	0
w_{AD}	0	0	0	0	3/4	-3/4
w_{BC}	0	0	0	0	1/4	1/4
w_{BD}	0	0	1/2	1/2	0	0
w_{CD}	1/4	1/4	0	0	0	0

Supplementary Table 5. Integer-valued tight-binding coefficients of the general simulation and experiment models for transmission line networks.

s_{XX} coefficient

	$-q_{14}$	-1	$-q_{1234}=-(q_{12}q_{34})$	$q_{1234}=(-q_{13}q_{24})$	$-q_{1234}=-(q_{14}q_{23})$
s_{AA}	-4	-2	-4	-2	0
s_{BB}	-2	-2	-4	-2	0
s_{CC}	2	-2	4	0	0
s_{DD}	4	2	4	0	0

v_{XX} coefficient

	$-q_{14}$	-1	$-q_{1234}=-(q_{12}q_{34})$	$q_{1234}=(-q_{13}q_{24})$	$-q_{1234}=-(q_{14}q_{23})$
v_{AA}	1	-1	-1	1	1
v_{BB}	-2	0	1	-1	-2
v_{CC}	2	1	1	1	2
v_{DD}	-1	0	-1	-1	-1

w_{XY} coefficient

	$-q_{14}$	-1	$-q_{1234}=-(q_{12}q_{34})$	$q_{1234}=(-q_{13}q_{24})$	$-q_{1234}=-(q_{14}q_{23})$
w_{AB}	1	1	1	1	1
w_{AC}	0	0	0	0	0
w_{AD}	0	0	0	0	0
w_{BC}	1	1	1	1	1
w_{BD}	0	0	0	0	0
w_{CD}	1	1	1	1	-1

Supplementary Note 4. Analytical solutions of edge states for the flat-band models

Here we present an analytic method to find the exact solutions of edge states for the flat-band models. We consider the following five types: (i) Edge states of the charges $\pm q_{mn}$; (ii) Edge states of the charges $\pm q_{1234}$; (iii) Evolution of edge states of the charges $\pm q_{1234}$ between different factorizations; (iv) Edge states of the charge -1 represented by q_{mn}^2; (v) Evolution of edge states of the charge -1.

(i) Edge states of the charges ±q_{mn}

The 1D Hamiltonian corresponding to the above charges can be constructed as $H(k) = R(k)diag(1,2,3,4)R^T(k)$, where the rotation matrix $R(k) = e^{\frac{k}{2}L_{ij}}$ with $k = 0 \to 2\pi$ and $i, j = 1,2,3,4$. It should be noted that the choice of $e^{\frac{k}{2}L_{ij}}$ is different from the choice of $e^{\frac{k+\pi}{2}L_{ij}}$ with $k = -\pi \to \pi$ (used in the main text). The eigen energy of edge states is independent of the choice. In order to find the edge states of the system, we rewrite $H(k)$ in the form of,

$$H(k) = H_{11} + H_{12}^* e^{-ik} + H_{12} e^{ik} \tag{16}$$

Take the charge q_{34} as an example. We have,

$$H_{11} = \begin{pmatrix} 1 & 0 & 0 & 0 \\ 0 & 2 & 0 & 0 \\ 0 & 0 & \frac{7}{2} & 0 \\ 0 & 0 & 0 & \frac{7}{2} \end{pmatrix} \tag{17}$$

and,

$$H_{12}^* = \frac{1}{4}\begin{pmatrix} 0 & 0 & 0 & 0 \\ 0 & 0 & 0 & 0 \\ 0 & 0 & -1 & -i \\ 0 & 0 & -i & 1 \end{pmatrix} \tag{18}$$

In terms of the tight-binding model, the diagonal elements of H_{11} describe the site energies and H_{12}^* and H_{12} describe the nearest-neighbour hoppings between two sites. Since all bulk modes are strongly localized in real space due to the fact that all bands are flat, it is natural to assume that the edge modes of the system are also strongly localized at the boundaries. For a system of N sites, we use the following ansatz for the edge state wave functions: $\Psi_L = (a, b, c, d)^T \delta_{n,1}$

and $\Psi_R = (a', b', c', d')^T \delta_{n,N}$, for the left and right edge states, respectively. To study the edge state at the left end, we let N goes to infinity. Since the wave function vanishes for all sites except $n = 1$, the only non-trivial equations of motion at sites $n = 1$ and 2 we need to consider are $(H_{11} - E)(a, b, c, d)^T = 0$ and $H_{12}^*(a, b, c, d)^T = 0$, respectively. It is easy to see that the matrix H_{12}^* is defective and there exists only one solution for the edge state: $\Psi_L = (0,0,1, i)^T \delta_{n,1}$ and $E = \frac{7}{2}$. Similarly, for the edge state at the right boundary, we find from the equations of motion at sites $n = N$ and $N - 1$, i.e., $(H_{11} - E)(a', b', c', d')^T = 0$ and $H_{12}(a', b', c', d')^T = 0$, the solution $\Psi_R = (0,0,1, -i)^T \delta_{n,N}$ and $E = \frac{7}{2}$. Thus two edge states are degenerate and have their wave functions in complex conjugate pairs. This is the result of PT symmetry. In the following, we will only consider the left edge state. The method can be equally applied to all other $\pm q_{mn}$ charges in this subsection. For charge $-q_{34}$, we only need to replace k by $-k$ in $H(k)$. Since the solutions found here satisfy the equations of motion for all sites, they are exact solutions. Here we do not concern with the normalization of the wave function.

(ii) Edge states of the charges ±q1234

This charge q_{1234} can be factorized into three different configurations, i.e., $q_{12}q_{34}$, $-q_{13}q_{24}$ and $q_{14}q_{23}$. The corresponding rotation matrices are $R(k) = e^{kL_{12}/2}e^{kL_{34}/2}$, $e^{-kL_{13}/2}e^{kL_{24}/2}$ and $e^{kL_{14}/2}e^{kL_{23}/2}$, respectively, from which we can obtain $H(k)$ for each configuration. By expressing $H(k)$ in terms of H_{11} and H_{12}^* and solving the equations of motion at the sites $n = 1$ and 2, i.e., $(H_{11} - E)(a, b, c, d)^T = 0$ and $H_{12}^*(a, b, c, d)^T = 0$, we find two edge states for each configuration, i.e., $E = \frac{3}{2}$ with $\Psi_L = (1, i, 0,0)^T \delta_{n,1}$ and $E = \frac{7}{2}$ with $\Psi_L = (0,0, 1, i)^T \delta_{n,1}$ for the case of $q_{12}q_{34}$; $E = 2$ with $\Psi_L = (1,0, -i, 0)^T \delta_{n,1}$ and $E = 3$ with $\Psi_L = (0,1, 0, i)^T \delta_{n,1}$ for the case of $-q_{13}q_{24}$; $E = \frac{5}{2}$ with $\Psi_L = (1,0,0, i)^T \delta_{n,1}$ and $E = \frac{5}{2}$ with $\Psi_L = (0,1, i, 0)^T \delta_{n,1}$ for the case of $q_{14}q_{23}$.

(iii) Evolution of edge states of the charges ±q1234 between different factorizations

Here we first consider the trajectory of edge states when the system is continuously transformed from one configuration to another one. There are three possible cases: (a) From $q_{12}q_{34}$ to $-q_{13}q_{24}$; (b) From $-q_{13}q_{24}$ to $q_{14}q_{23}$; (c) From $q_{14}q_{23}$ to $q_{12}q_{34}$.

For case (c), we consider two commuting hybrid generators $L_a = cos\theta L_{14} + \sin\theta L_{12}$ and $L_b = \cos\theta\, L_{23} + \sin\theta L_{34}$, where the parameter $\theta \in \left[0, \frac{\pi}{2}\right]$ describing the transition from $q_{14}q_{23}$ to $q_{12}q_{34}$. From the corresponding rotation matrix $R(k,\theta) = e^{kL_a/2}e^{kL_b/2}$, we obtain the Hamiltonian $H(k,\theta) = R(k,\theta) diag(1,2,3,4) R^T(k,\theta)$ and the decomposed components:

$$H_{11} = \begin{pmatrix} 2+\frac{1}{2}\cos 2\theta & 0 & \frac{1}{2}\sin 2\theta & 0 \\ 0 & 2+\frac{1}{2}\cos 2\theta & 0 & -\frac{1}{2}\sin 2\theta \\ \frac{1}{2}\sin 2\theta & 0 & 3-\frac{1}{2}\cos 2\theta & 0 \\ 0 & -\frac{1}{2}\sin 2\theta & 0 & 3-\frac{1}{2}\cos 2\theta \end{pmatrix} \tag{19}$$

$$H_{12}^{*} = \frac{1}{4}\begin{pmatrix} -2-\cos 2\theta & -i\sin\theta & -\sin 2\theta & -3i\cos\theta \\ -i\sin\theta & -\cos 2\theta & -i\cos\theta & \sin 2\theta \\ -\sin 2\theta & -i\cos\theta & \cos 2\theta & -i\sin\theta \\ -3i\cos\theta & \sin 2\theta & -i\sin\theta & 2+\cos 2\theta \end{pmatrix} \tag{20}$$

The matrix H_{12}^{*} is partially defective and $H_{12}^{*}\varphi = 0$ has two eigenvectors, which can be chosen as $\varphi_1 = (-i, \sin\theta, 0, \cos\theta)^T$ and $\varphi_2 = (-\sin\theta, -i, \cos\theta, 0)^T$. We write the edge state wave functions as $c_1\varphi_1 + c_2\varphi_2$ and solve the equation $(H_{11} - E)(c_1\varphi_1 + c_2\varphi_2) = 0$ for E and $c_{1,2}$. We find the two edge states at energies $E^{\pm} = \frac{5}{2} \pm \sin\theta$. This result is shown in Fig. 3f. The corresponding wave functions are $\Psi_L^{\pm} = [\mp i\sec\theta\,\varphi_1 - \sec\theta\,\varphi_2]\delta_{n,1}$. The edge state energies can also be obtained directly from the eigenvalues of H_{11} without knowing the edge state wave functions. Similarly, we apply the same procedure to cases (a) and (b) and find the following trajectories of the edge state energies: $E^{\pm} = \frac{5}{2} \pm \frac{\sqrt{2}}{4}\sqrt{5+3\cos 2\theta}$ for case (a) and $E^{\pm} = \frac{5}{2} \pm \frac{1}{2}\cos\theta$ for case (b). These results are plotted in Figs. 3d and 3e, respectively.

(iv) Edge states of the charge -1 represented by q_{mn}^2

For the above cases, the rotation matrix becomes $R(k) = e^{kL_{ij}}$. We can simply replace k in $H(k)$ by $2k$ and rewrite it in the form of $H(k) = H_{11} + H_{13}^{*}e^{-2ik} + H_{13}e^{2ik}$. In the real space, such replacement represents a next-nearest neighbor hopping. For a finite chain with even number of sites, the tight-binding Hamiltonian becomes two disconnected sublattices, one with indices $n = 1,3,5 \ldots N-1$ and the other with $n = 2,4,6 \ldots N$. For the case of q_{34}^2, the matrices H_{11} and H_{13}^{*} are identical to those shown in Supplementary Eq. (17) and (18), respectively. From which we find one edge state at the left boundary per sublattice, i.e., $\Psi_{L1} = (0,0,1,i)^T\delta_{n,1}$ and $\Psi_{L2} = (0,0,1,i)^T\delta_{n,2}$ with the same eigen energy at $E = \frac{7}{2}$.

(v) Evolution of edge states of the charge -1

Here we consider the following three cases: (a) Transition from q_{ij}^2 to q_{jk}^2; (b) Edge state evolution involving the mixing of q_{ij}^2, q_{jk}^2 and q_{ik}^2 with rotation of eigenvectors in three bands; (c) Edge state evolution involving the mixing of q_{ij}^2, q_{jk}^2 and q_{jl}^2 with rotation of eigenvectors in four bands.

(a) Transition from q_{ij}^2 to q_{jk}^2

As an example, we consider a continuous transition from q_{23}^2 to q_{12}^2. We choose a hybrid generator $L = \cos t\, L_{23} + \sin t\, L_{12}$ with $t \in \left[0, \frac{\pi}{2}\right]$. We obtain the Hamiltonian through $H(k,t) = R(k,t) diag(1,2,3,4) R^T(k,t)$ and rewrite it in the form of,

$$H(k,t) = H_{11}(t) + H_{12}^*(t)e^{-ik} + H_{12}(t)e^{ik} + H_{13}^*(t)e^{-2ik} + H_{13}(t)e^{2ik} \quad (21)$$

where,

$$H_{11}(t) = \frac{1}{8}\begin{pmatrix} -2\,cos(2t) - 3\,cos(4t) + 13 & 0 & -3\,sin(4t) & 0 \\ 0 & 4(cos(2t)+4) & 0 & 0 \\ -3\,sin(4t) & 0 & -2\,cos(2t) + 3\,cos(4t) + 19 & 0 \\ 0 & 0 & 0 & 32 \end{pmatrix} \quad (22)$$

$$H_{12}^* = -\frac{\sin(2t)}{2}\begin{pmatrix} \sin(2t) & i\cos(t) & -\cos(2t) & 0 \\ i\cos(t) & 0 & i\sin(t) & 0 \\ -\cos(2t) & i\sin(t) & -\sin(2t) & 0 \\ 0 & 0 & 0 & 0 \end{pmatrix} \quad (23)$$

$$H_{13}^* = \frac{\cos(2t)}{4}\begin{pmatrix} \sin^2(t) & i\sin(t) & -\cos(t)\sin(t) & 0 \\ i\sin(t) & -1 & -i\cos(t) & 0 \\ -\cos(t)\sin(t) & -i\cos(t) & \cos^2(t) & 0 \\ 0 & 0 & 0 & 0 \end{pmatrix} \quad (24)$$

At $t = 0$ or $\frac{\pi}{2}$, $H_{12} = 0$ and the system reduces to two disconnected sublattices and H_{13} describes the hopping elements within each sublattice. For a general value of t, H_{12} couples two sublattices and it is natural to assume that an edge state occupies two sites at boundary, one from each sublattice. Thus, we use the edge state ansatz with the form of $\Psi_L = \Phi_1\delta_{n,1} + \Phi_2\delta_{n,2}$, where $\Phi_1 = (a,b,c,d)^T$ and $\Phi_2 = (e,f,g,h)^T$. Both the wave function and the edge state energy E are to be determined through solving the equations of motion. Since the wave function vanishes for sites with $n > 2$, we only need to consider the equations of motion of the four boundary sites, i.e.,

$$(H_{11} - E)\Phi_1 + H_{12}\Phi_2 = 0 \quad (n = 1) \quad (25)$$

$$(H_{11} - E)\Phi_2 + H_{12}^*\Phi_1 = 0 \quad (n = 2) \quad (26)$$

$$H_{13}^*\Phi_1 + H_{12}^*\Phi_2 = 0 \quad (n = 3) \quad (27)$$

$$H_{13}^*\Phi_2 = 0 \quad (n = 4) \quad (28)$$

Both the matrices H_{13}^* and H_{12}^* are defective. H_{13}^* has two independent eigen vectors, which can be chosen as $(\sin t, i, -\cos t, 0)^T$ and $(\cos t, 0, \sin t, 0)^T$. However, the matrix H_{12}^* has only one coalesced eigenvector in the form of $(\sin t, i, -\cos t\,, 0)^T$. With these eigen vectors of H_{13}^* and H_{12}^*, a simple edge state can be obtained immediately by choosing $\Phi_2 = 0$ and $\Phi_1 = (\sin t, i, -\cos t\,, 0)^T$, which satisfies Supplementary Eq. (25-28). Eigenvalue of such an edge state is determined from Supplementary Eq. (25). From the equation $(H_{11} - E)\Phi_1 = 0$, we obtain the first solution of the edge state,

$$E^0 = 2 + \frac{1}{2}\cos 2t \tag{29}$$

with,

$$\Psi_L^0 = (\sin t, i, -\cos t\,, 0)^T \delta_{n,1} \tag{30}$$

The other edge state solutions can be obtained by choosing $\Phi_2 = (\sin t, i, -\cos t, 0)^T$ and $\Phi_1 = c(\cos t, 0, \sin t\,, 0)^T$ so that Supplementary Eq. (27) and (28) are satisfied automatically. The unknown function c and the eigenvalue E are to be determined by solving Supplementary Eq. (25) and (26), from which we find two more edge states:

$$E^{\pm} = \frac{1}{8}\left(16 - 2\cos 2t \pm \sqrt{2}\sqrt{17 + \cos 4t}\right) \tag{31}$$

with,

$$\Psi_L^{\pm} = c_{\pm}(\cos t, 0, \sin t\,, 0)^T \delta_{n,1} + (\sin t, i, -\cos t\,, 0)^T \delta_{n,2} \tag{32}$$

where $c_{\pm} = \frac{-4\sin 2t}{3\cos 2t \pm \sqrt{9 - \sin^2 2t}}$. Supplementary Eq. (29) and (31) are plotted in the Panel "From q_{23}^2 to q_{12}^2" of Supplementary Figure 10c with $\theta_{23\to 12} = t$.

(b) Edge state evolution involving the mixing of q_{ij}^2, q_{jk}^2 and q_{ik}^2 with rotation of eigenvectors in three bands

Now we consider a more general case where the edge states form surfaces in a 2D parameter space. As an example, we consider a hybrid generator of the form:

$$L = \cos t\, L_{23} + \sin t \cos f\, L_{12} + \sin t \sin f\, L_{13} \tag{33}$$

where the parameters $t, f \in \left[0, \frac{\pi}{2}\right]$. From the rotation matrix $R(k,t) = e^{kL}$, we obtain the Hamiltonian $H(k,t,f)$ and the decomposed components of H_{11}, H_{12}^* and H_{13}^*. By solving Supplementary Eq. (25-28) we can obtain both the edge state energies and wave functions. However, the edge state energies can also be obtained from the roots of the determinant in Supplementary Eq. (25) and (26), i.e., $\det\begin{pmatrix} H_{11} - EI_4 & H_{12} \\ H_{12}^* & H_{11} - EI_4 \end{pmatrix} = 0$. From which, we find

four pairs of degenerate eigen energies. Apart from the trivial one at $E = 4$, the other three are edge state energies of the left and right edge states with the forms of,

$$E^0 = \frac{1}{16}(\cos(2f - 2t) + \cos(2f + 2t) - 2\cos(2f) + 6\cos(2t) + 34) \tag{34}$$

$$E^{\pm} = \frac{1}{32}\begin{pmatrix} -\cos(2f - 2t) - \cos\big(2(f + t)\big) + 2\cos(2f) - 6\cos(2t) + 62 \pm \\ \sqrt{8\cos(4f)\sin^4(t) - 48\cos(2f)\sin^2(t)(\cos(2t) - 5) + 148\cos(2t) + 19\cos(4t) + 409} \end{pmatrix} \tag{35}$$

Supplementary Eq. (34) and (35) are plotted in the Supplementary Figure 11a, which shows three surfaces of edge states involving three bands only. Since these edge states do not involve the fourth band, Supplementary Figure 11a also describes the edge state evolution of the charge -1 in the 3-band model[5] involving the mixtures of factorizations i^2, $j^2$and k^2.

It should be noted that Supplementary Figure 11a shows the edge state surfaces inside the triangle formed by q_{12}^2, q_{23}^2 and q_{13}^2 shown in Supplementary Figure 10a and b. At $f = 0$, Supplementary Eq. (34) and (35) reduce to Supplementary Eq. (29) and (31), describing the edge state evolution from q_{23}^2 to q_{12}^2. At $t = \frac{\pi}{2}$, the parameter f describes the evolution from q_{12}^2 to q_{13}^2 as can be seen from Supplementary Eq. (33). The above results reduce to,

$$E^0 = \frac{1}{4}(7 - \cos(2f)) \tag{36}$$

$$E^{\pm} = \frac{1}{8}\left(17 + \cos(2f) \pm \sqrt{2}\cos f\sqrt{\cos(2f) + 17}\right) \tag{37}$$

Supplementary Eq. (36) and (37) are plotted in the Panel "From q_{12}^2 to q_{13}^2" of Supplementary Figure 10c with $\theta_{12\to13} = f$.

At $f = \frac{\pi}{2}$, the parameter t describes the evolution from q_{23}^2 to q_{13}^2 as can be seen from Supplementary Eq. (33). Supplementary Eq. (34) and (35) reduce to,

$$E^0 = \frac{1}{4}(9 + \cos(2t)) \tag{38}$$

$$E^{\pm} = \frac{1}{8}\left(15 - \cos(2t) \pm \sqrt{2}\cos t\sqrt{\cos(2t) + 17}\right) \tag{39}$$

Supplementary Eq. (38) and (39) are plotted in the Panel "From q_{23}^2 to q_{13}^2" of Supplementary Figure 10c with $\theta_{23\to13} = t$. It should be noted the same analytic method can be applied to all edges of the regular octahedron as shown in Supplementary Figure 10a and b. It can also be used to obtain the 3D maps of edge state surfaces resulting from the mixing of q_{ij}^2, q_{jk}^2 and q_{ik}^2 for any three bands i, j and k.

(c) Edge state evolution involving the mixing of q_{ij}^2, q_{jk}^2 and q_{jl}^2 with rotation of eigenvectors in four bands

Finally, we consider a hybrid generator of the form,

$$L = \cos t\, L_{12} + \sin t \cos f\, L_{13} + \sin t \sin f\, L_{14} \tag{40}$$

where $t, f \in \left[0, \frac{\pi}{2}\right]$. Different from case (b), Supplementary Eq. (40) now produces the edge state surfaces inside the triangle formed by q_{12}^2, q_{13}^2 and q_{14}^2 highlighted in the Supplementary Figure 10a and b, in which all four bands are involved. By using the same procedure of case (b), we obtain the four edge state surfaces as shown in Supplementary Figure 11b, which fits well with the numerical results shown in Supplementary Figure 10d. One of the four surfaces has the form of,

$$E^0 = \frac{1}{16}\left(\cos\left(2(f-t)\right) + \cos\left(2(f+t)\right) - 2\cos(2f) - 6\cos(2t) + 30\right) \tag{41}$$

The other three ones are complex as the roots of a cubic equation.

Thus, the method can be used to obtain the edge states inside every face triangle of the regular octahedron as shown in the Supplementary Figure 10a and b. The number of edge state surfaces depends on the number of bands involved.

Reference:

1 Wu, Q., Soluyanov, A. A. & Bzdušek, T. Non-Abelian band topology in noninteracting metals. *Science* **365**, 1273, doi:10.1126/science.aau8740 (2019).

2 Perez-Gracia, A. & Thomas, F. On Cayley's Factorization of 4D Rotations and Applications. *Advances in Applied Clifford Algebras* **27**, 523-538, doi:10.1007/s00006-016-0683-9 (2017).

3 Le Bihan, N. The geometry of proper quaternion random variables. *Signal Processing* **138**, 106-116, doi:https://doi.org/10.1016/j.sigpro.2017.03.017 (2017).

4 Erdoğdu, M. & Özdemir, M. Simple, Double and Isoclinic Rotations with a Viable Algorithm. *Mathematical Sciences and Applications E-Notes, 8 (1), 11-24*, doi:10.36753/mathenot.642208 (2020).

5 Guo, Q. *et al.* Experimental observation of non-Abelian topological charges and edge states. *Nature* **594**, 195-200, doi:10.1038/s41586-021-03521-3 (2021).

Supplementary Figures

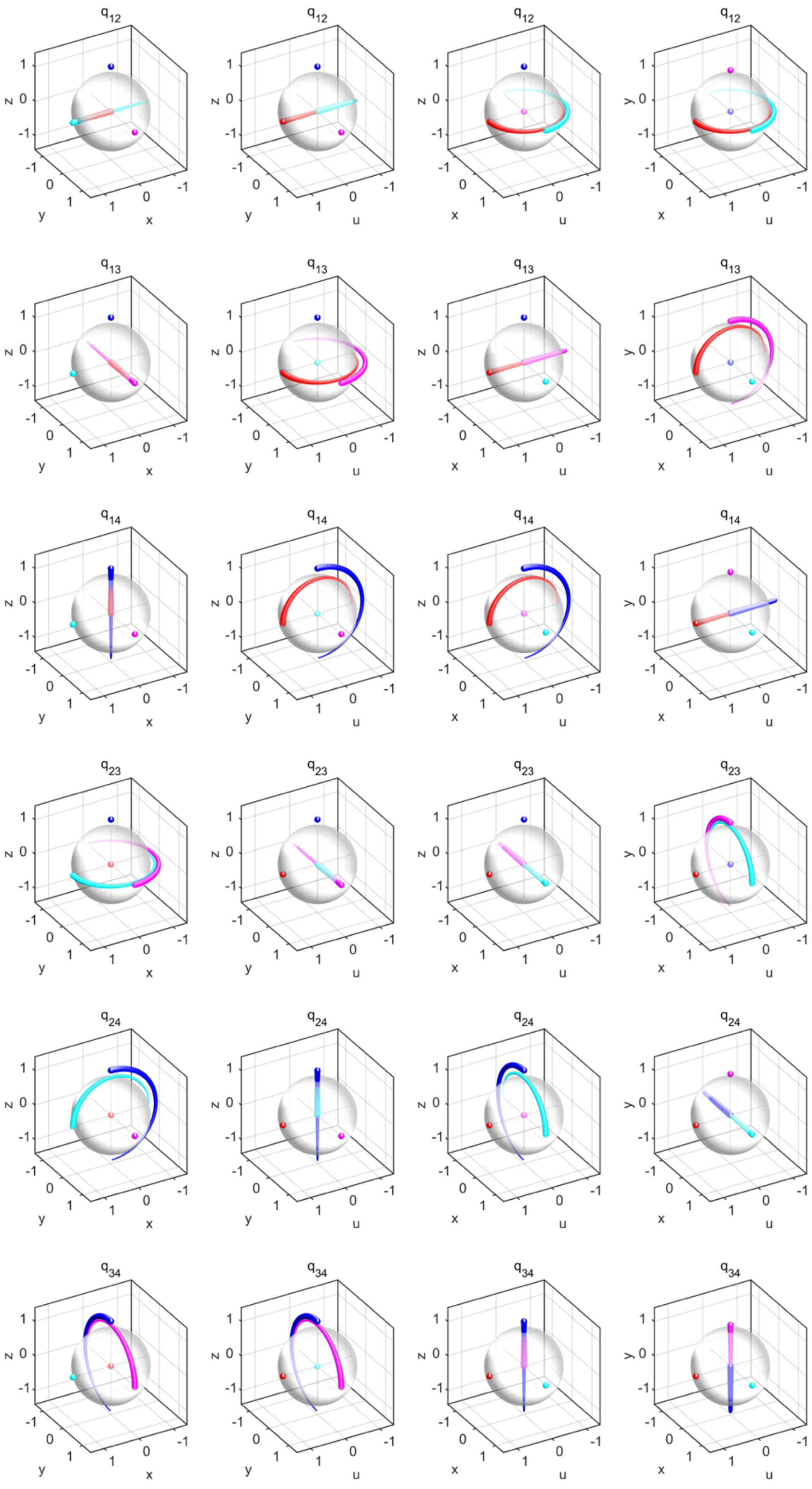
q12
q13
q14
q23
q24
q34
x
y
z
u

Supplementary Figure 1. Trajectories of eigenstates of charges q_{mn} orthographically projected onto four solid spheres in $\mathbb{R}^3$. The colours (red, cyan, magenta, blue) correspond to the (first, second, third, fourth) bands. The direction of line-width decreasing indicates $k = -\pi \rightarrow \pi$.

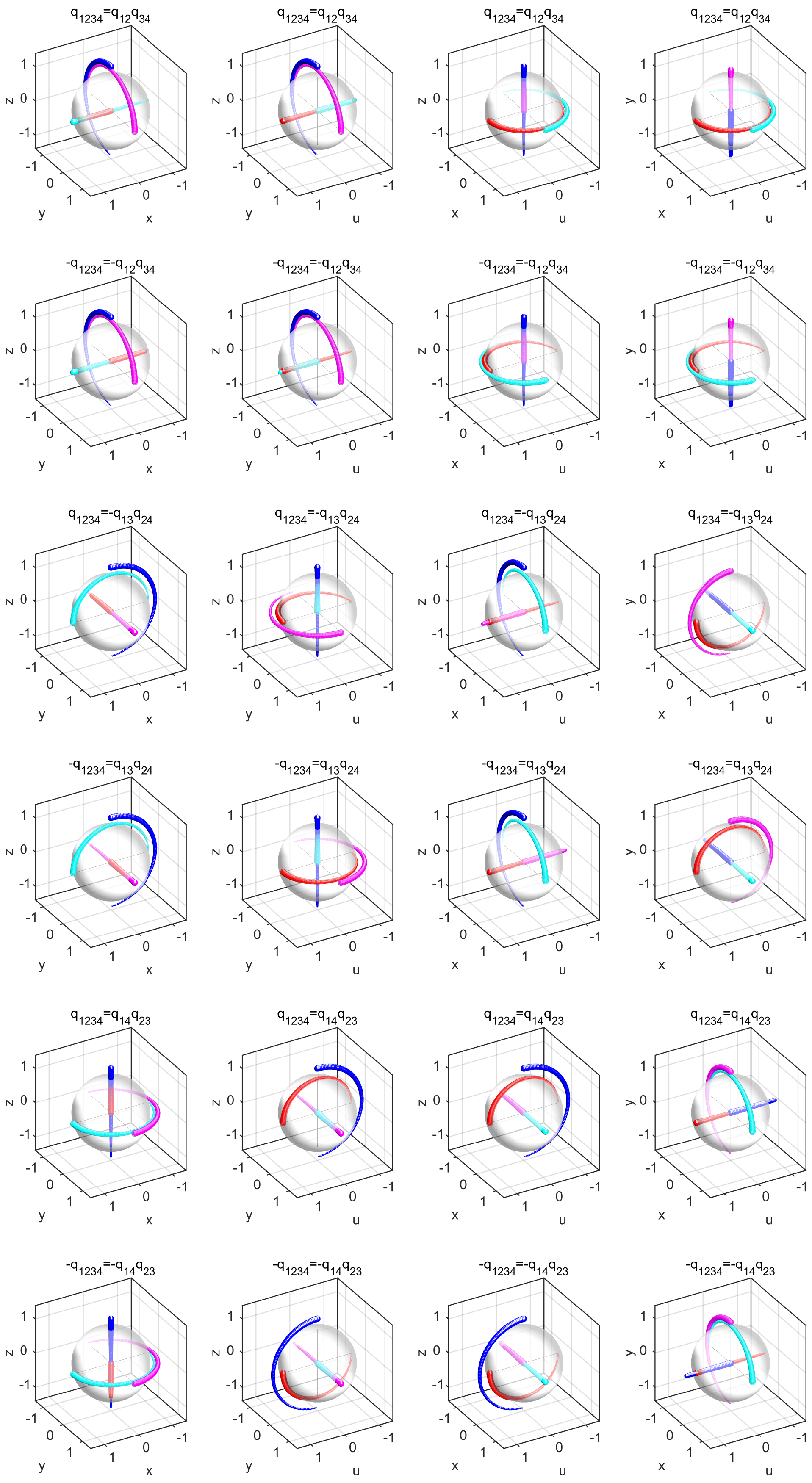
$q_{1234}=q_{12}q_{34}$
$q_{1234}=q_{12}q_{34}$
$q_{1234}=q_{12}q_{34}$
$q_{1234}=q_{12}q_{34}$
$-q_{1234}=-q_{12}q_{34}$
$-q_{1234}=-q_{12}q_{34}$
$-q_{1234}=-q_{12}q_{34}$
$-q_{1234}=-q_{12}q_{34}$
$q_{1234}=-q_{13}q_{24}$
$q_{1234}=-q_{13}q_{24}$
$q_{1234}=-q_{13}q_{24}$
$q_{1234}=-q_{13}q_{24}$
$-q_{1234}=q_{13}q_{24}$
$-q_{1234}=q_{13}q_{24}$
$-q_{1234}=q_{13}q_{24}$
$-q_{1234}=q_{13}q_{24}$
$q_{1234}=q_{14}q_{23}$
$q_{1234}=q_{14}q_{23}$
$q_{1234}=q_{14}q_{23}$
$q_{1234}=q_{14}q_{23}$
$-q_{1234}=-q_{14}q_{23}$
$-q_{1234}=-q_{14}q_{23}$
$-q_{1234}=-q_{14}q_{23}$
$-q_{1234}=-q_{14}q_{23}$

Supplementary Figure 2. Trajectories of eigenstates of charges $\pm q_{1234}$ orthographically projected onto four solid spheres in $\mathbb{R}^3$. We factorize the charge q_{1234} into three configurations: $q_{1234} = q_{12}q_{34}$, $q_{1234} = -q_{13}q_{24}$ and $q_{1234} = q_{14}q_{23}$. The colours (red, cyan, magenta, blue) correspond to the (first, second, third, fourth) bands. The direction of line-width decreasing indicates $k = -\pi \to \pi$.

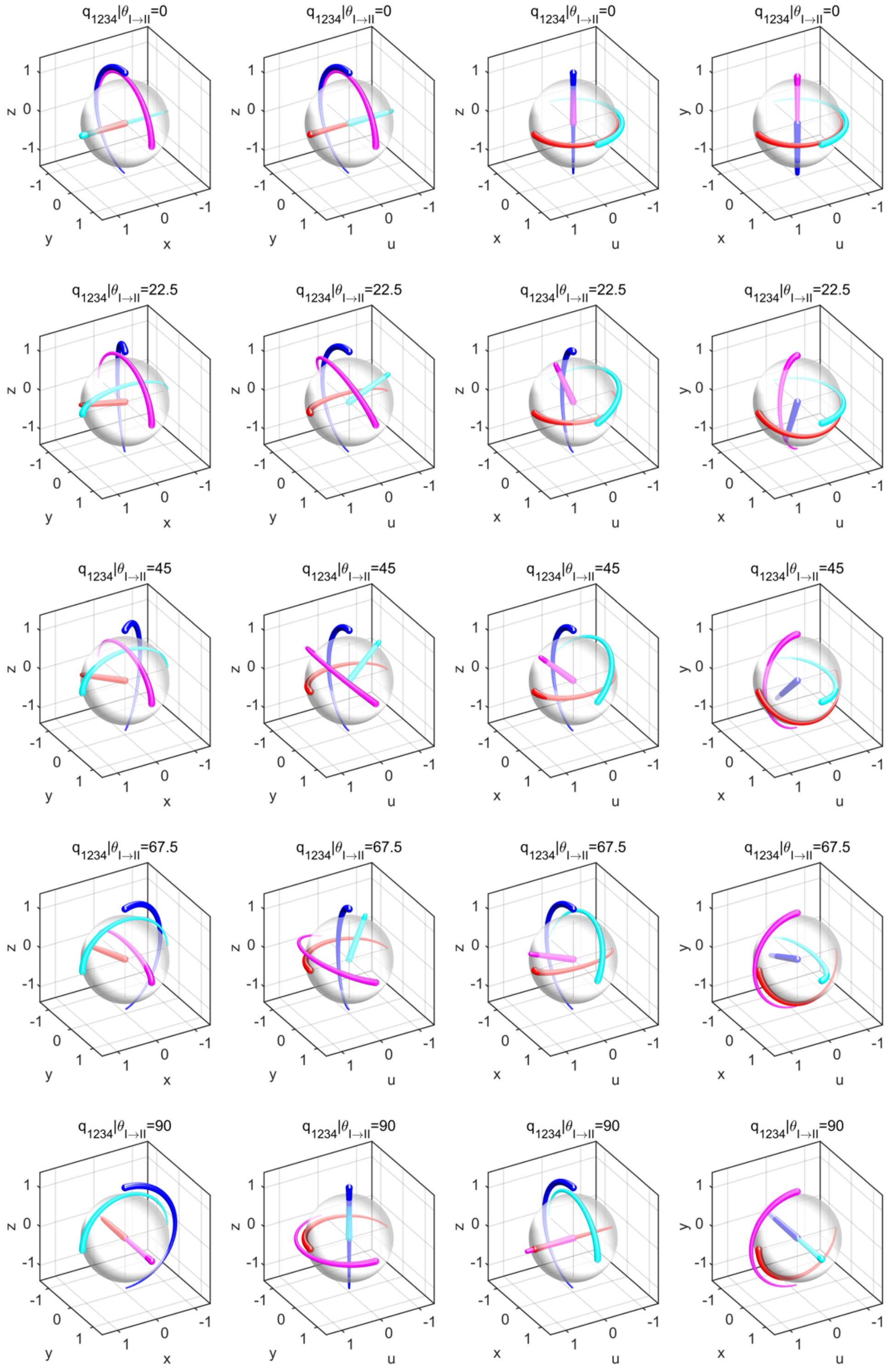

Supplementary Figure 3. Trajectories of eigenstates of charge q_{1234} orthographically projected onto four solid spheres in $\mathbb{R}^3$. We continuously rotate the charge q_{1234} from the configuration $q_{1234} = q_{12}q_{34}$ to $q_{1234} = -q_{13}q_{24}$, where the parameter $\theta_{I\to II}$ is defined as, $L_a = \cos\theta_{I\to II} L_{12} - \sin\theta_{I\to II} L_{13}$ *and* $L_b = \cos\theta_{I\to II} L_{34} + \sin\theta_{I\to II} L_{24}$. The Hamiltonian can be written as $H = \exp\left(\frac{k_\pi}{2} L_b\right) \exp\left(\frac{k_\pi}{2} L_a\right) I_{1234} \exp\left(-\frac{k_\pi}{2} L_a\right) \exp\left(-\frac{k_\pi}{2} L_b\right)$ with $k_\pi = k + \pi$.

The colours (red, cyan, magenta, blue) correspond to the (first, second, third, fourth) bands. The direction of line-width decreasing indicates $k = -\pi \rightarrow \pi$.

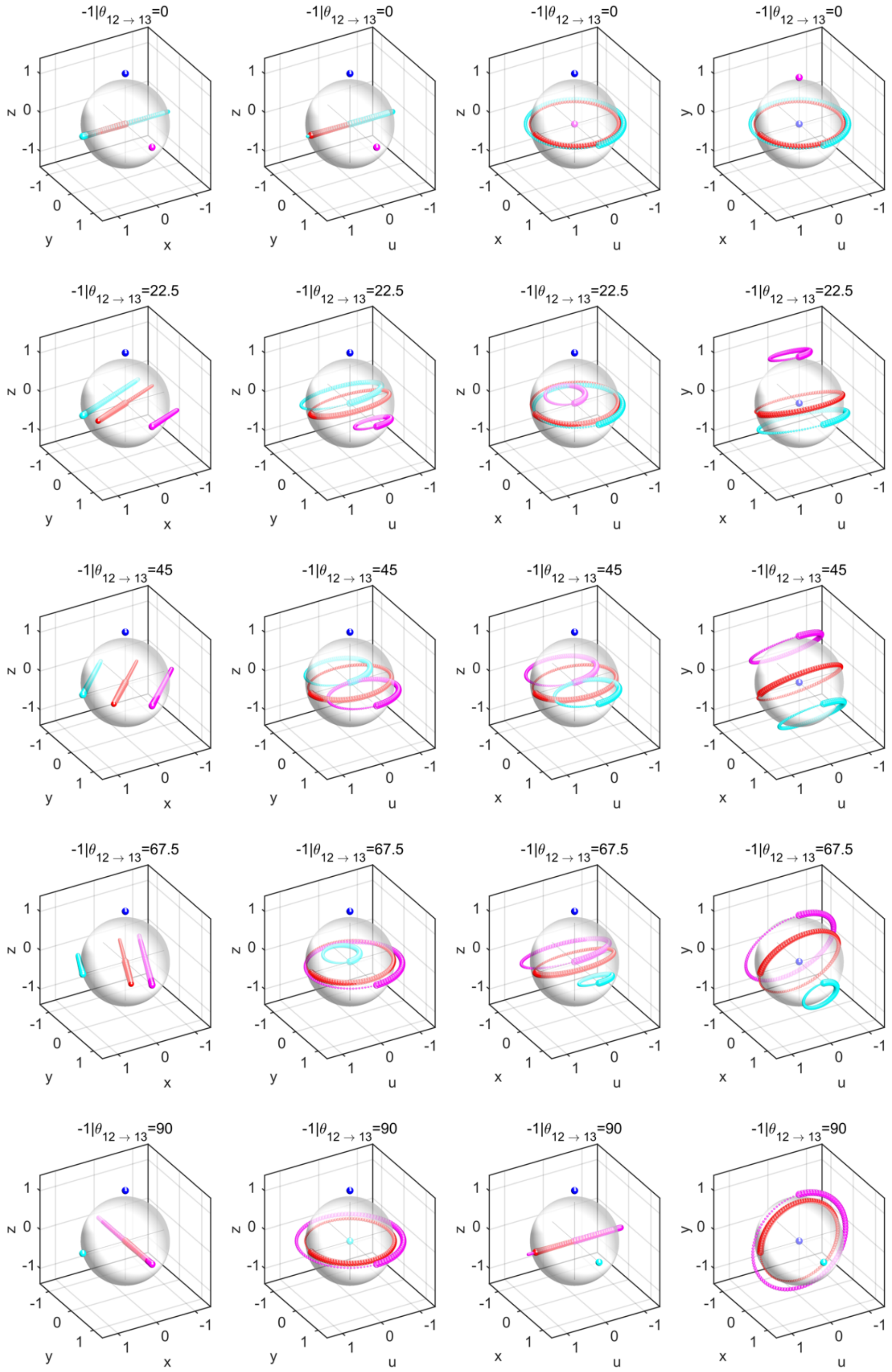

Supplementary Figure 4. Trajectories of eigenstates of charge -1 orthographically projected onto four solid spheres in $\mathbb{R}^3$. We continuously rotate the charge -1 from the configuration

$-1 = q_{12}^2$ to $-1 = -q_{13}^2$, where the parameter $\theta_{12\to13}$ is defined as, $L = \cos\theta_{12\to13} L_{12} + \sin\theta_{12\to13} L_{13}$. The Hamiltonian can be written as $H = \exp(k_\pi L)\, I_{1234} \exp(-k_\pi L)$ with $k_\pi = k + \pi$. The colours (red, cyan, magenta, blue) correspond to the (first, second, third, fourth) bands. The direction of line-width decreasing indicates $k = -\pi \to \pi$.

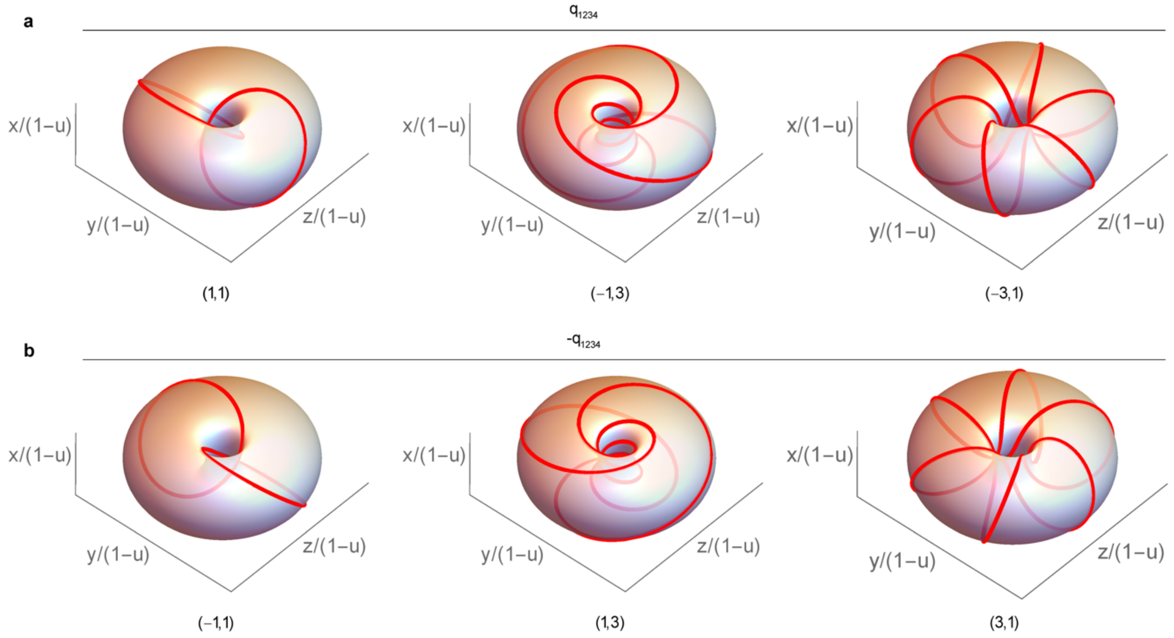

Supplementary Figure 5. Stereographically projected Clifford tori in $\mathbb{R}^3$, i.e. $(u,x,y,z) \to \left(\frac{x}{1-u}, \frac{y}{1-u}, \frac{z}{1-u}\right)$. The index (m,n) indicates the eigenstates rotate $m\pi$ and $n\pi$ on the oux and oyz planes, respectively. a, The three cases corresponding to charge $+q_{1234}$, they can be continuously transformed into each other. b, The opposite rotation senses corresponding to charge $-q_{1234}$.

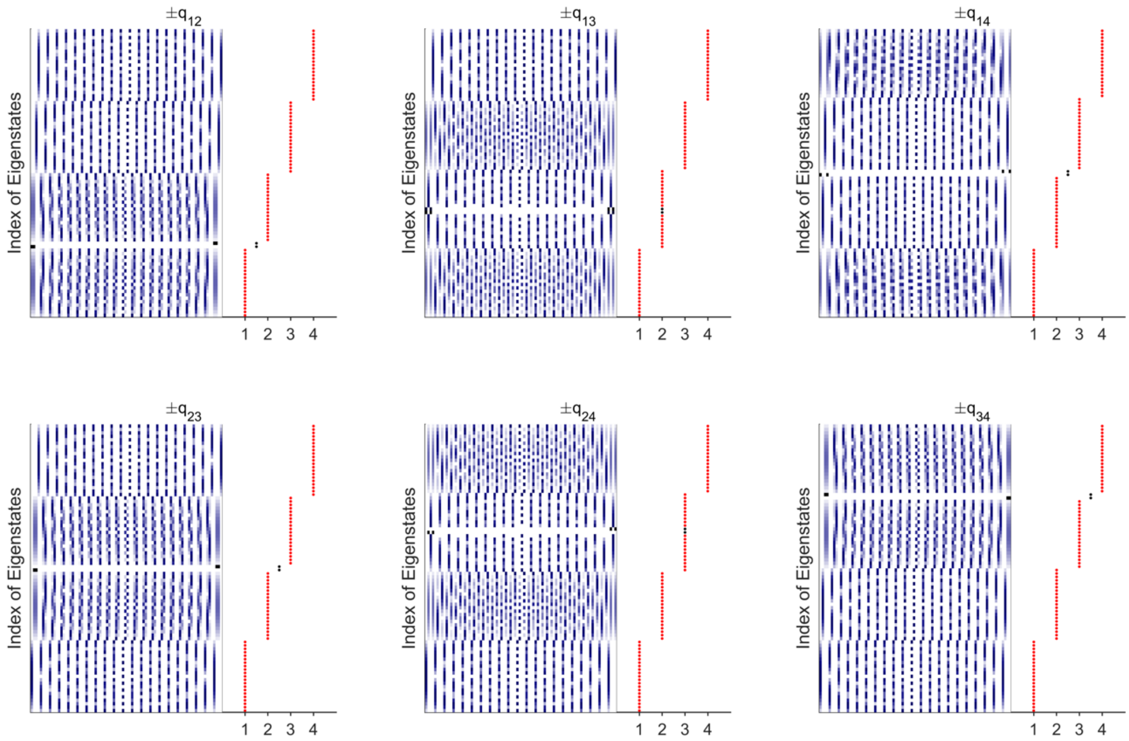

Supplementary Figure 6. Edge state distributions at the hard boundaries of a finite lattice for charges $\pm q_{mn}$ of flat band models.

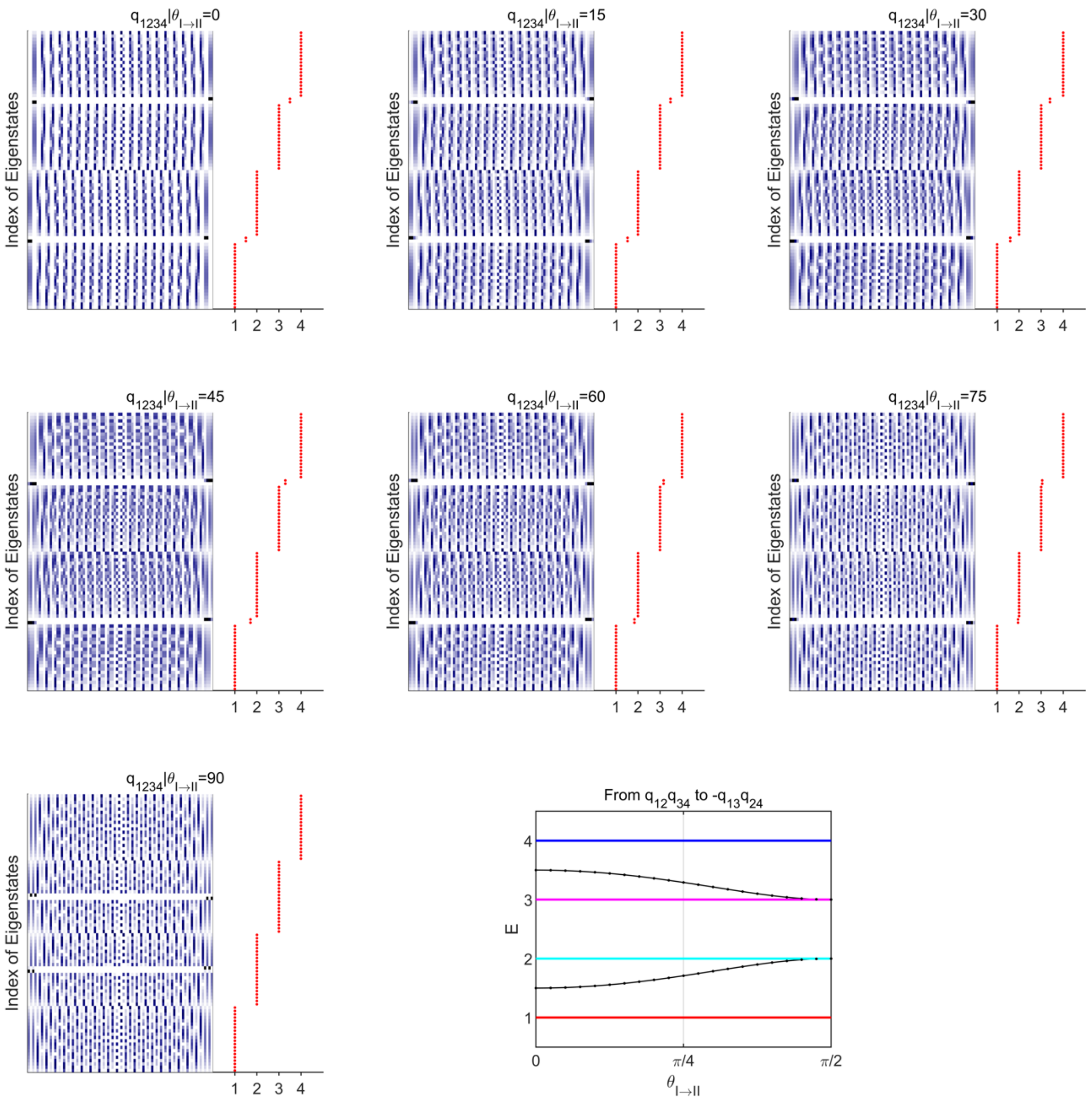

Supplementary Figure 7. Evolution of edge state distributions for charge q_{1234} from the factorization of $q_{1234} = q_{12}q_{34}$ to $q_{1234} = -q_{13}q_{24}$, parametrized by $\theta_{I\to II}$ with unit of degrees. Lines/dots indicate numerical/analytical results.

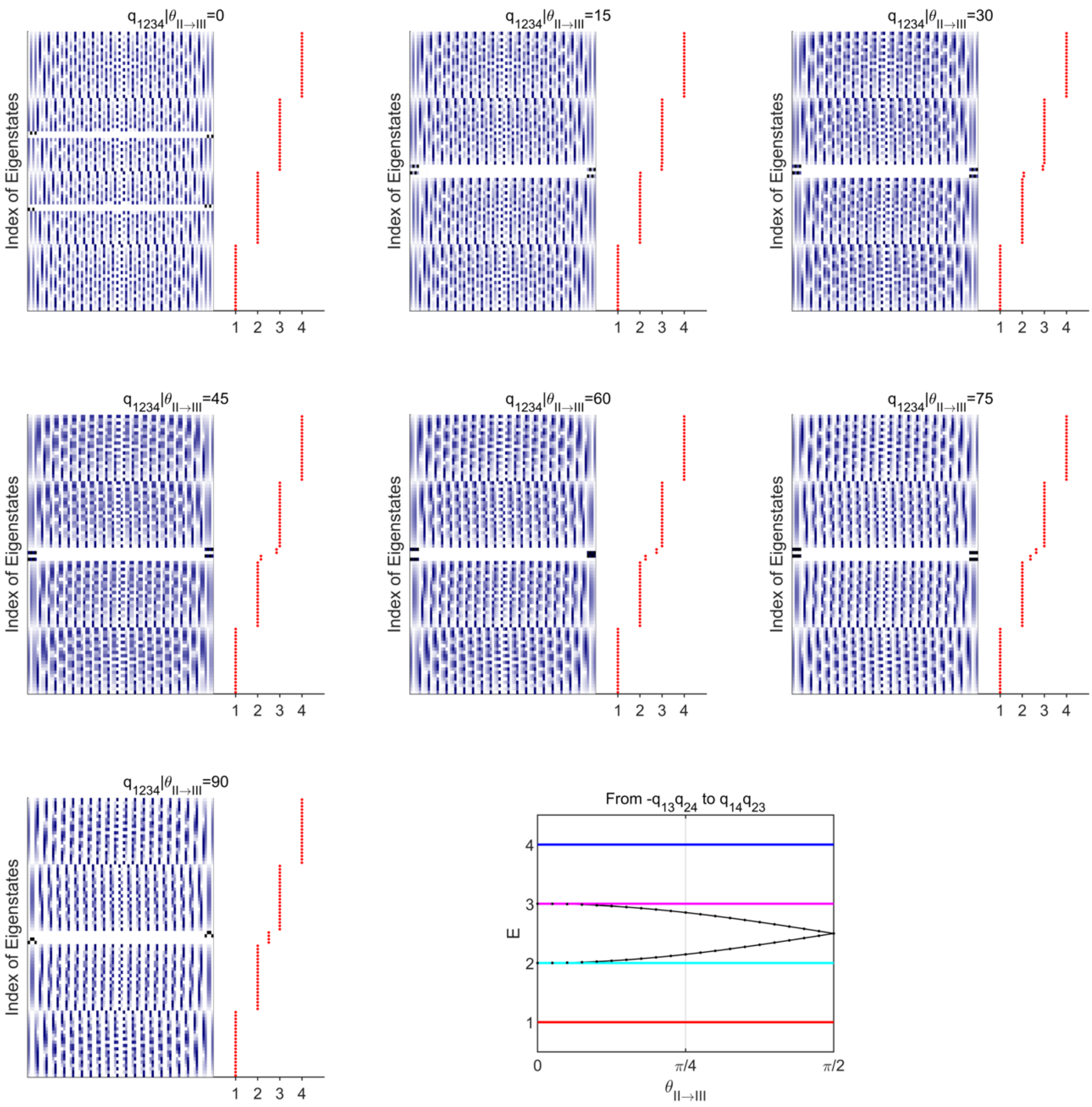

Supplementary Figure 8. Evolution of edge state distributions for charge q_{1234} from the factorization of $q_{1234} = -q_{13}q_{24}$ to $q_{1234} = q_{14}q_{23}$, parametrized by $\theta_{II\to III}$ with unit of degrees. Lines/dots indicate numerical/analytical results.

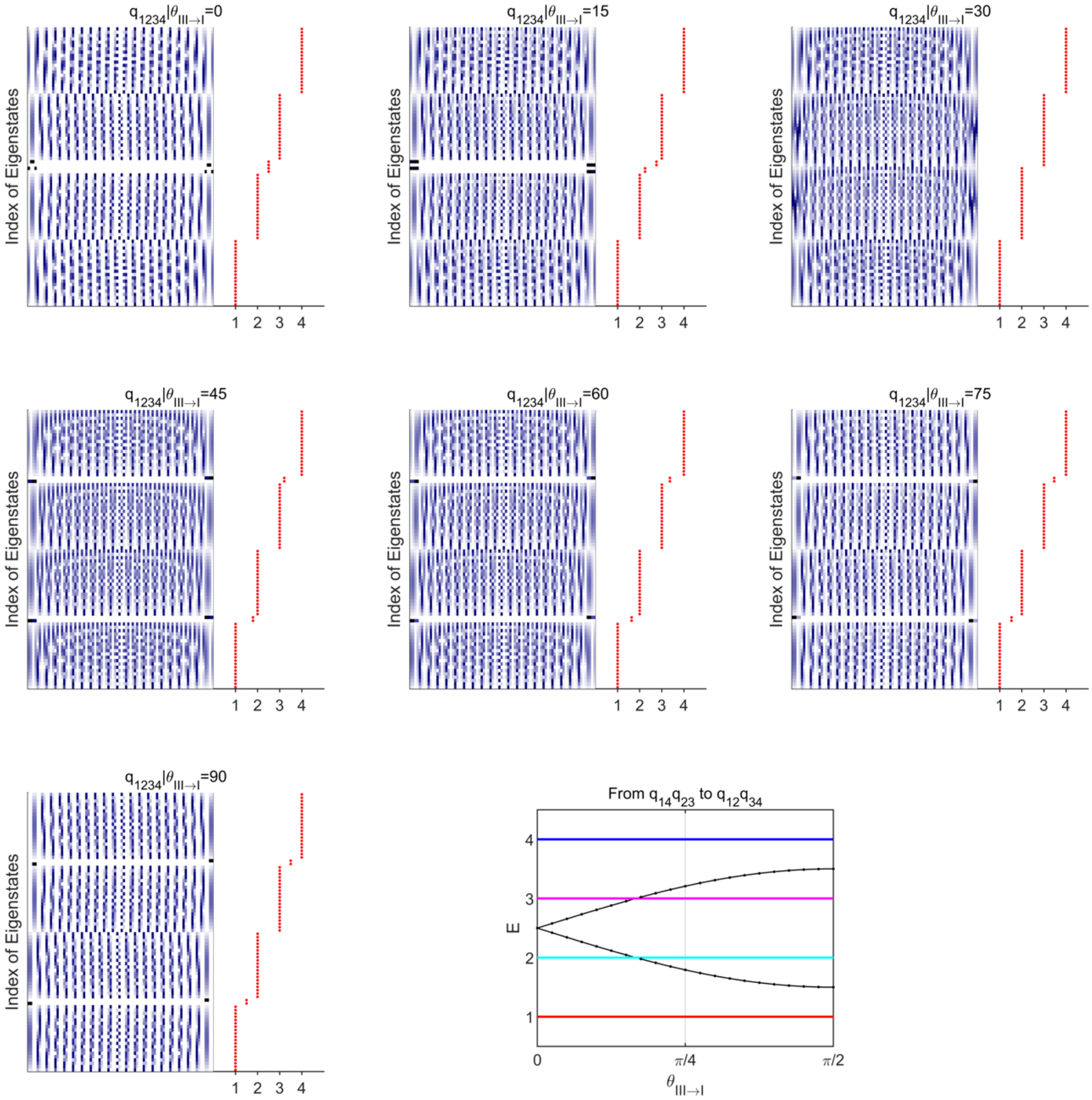

Supplementary Figure 9. Evolution of edge state distributions for charge q_{1234} from the factorization of $q_{1234} = q_{14}q_{23}$ to $q_{1234} = q_{12}q_{34}$, parametrized by $\theta_{III \to I}$ with unit of degrees. Lines/dots indicate numerical/analytical results.

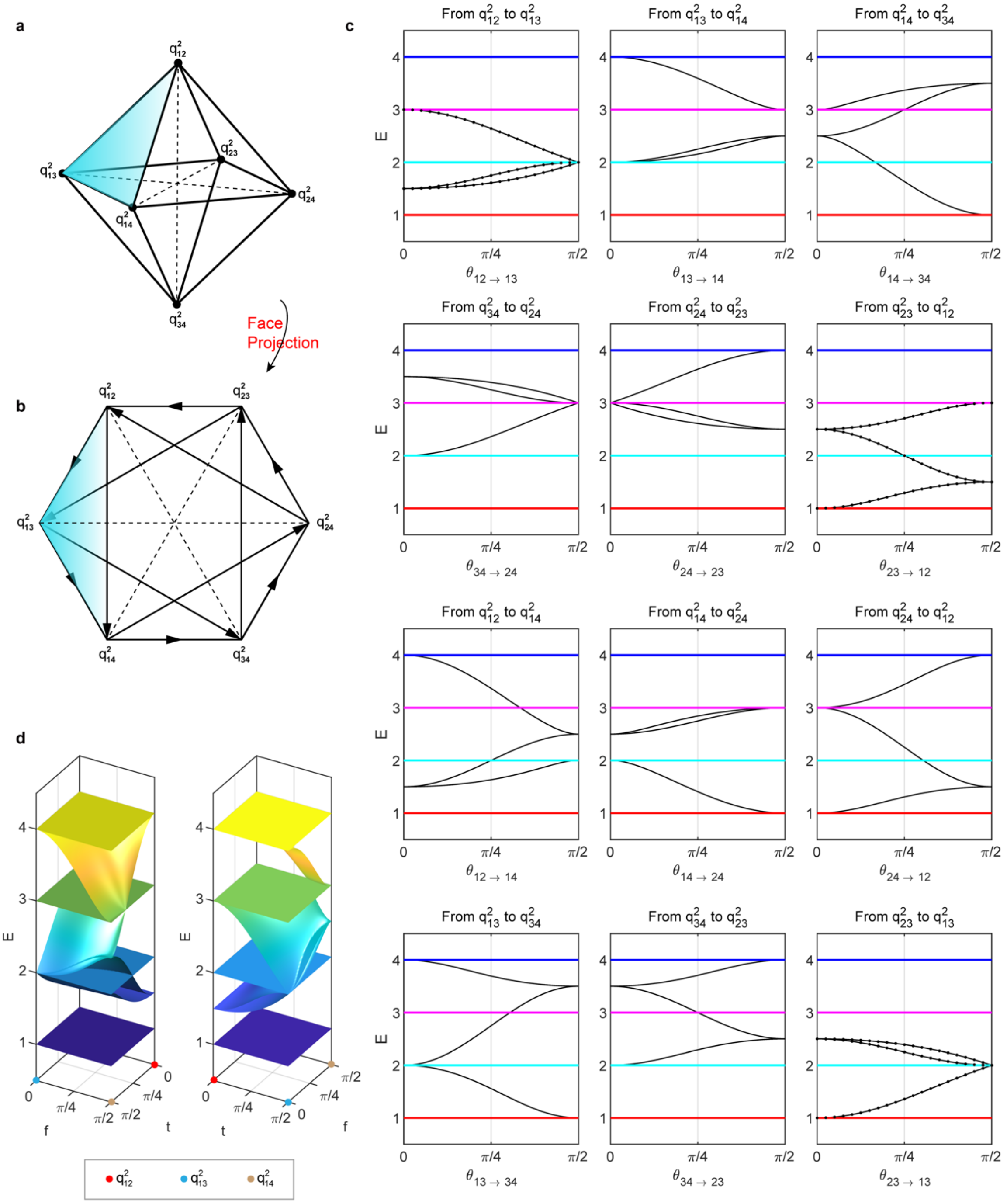

Supplementary Figure 10. Evolution of edge state distributions for charge −1. **a,** All possible factorizations of charge −1 illustrated on a regular octahedron. **b,** Orthogonal projection centred by face. There are 12 possible transitions, the direct transition (dashed lines) between one pair of diagonal points is not allowed as they are located on two orthogonal planes, i.e. $q_{12}^2 \not\Leftrightarrow q_{34}^2$. **c,** Evolution of edge state distributions along 12 edges of the regular octahedron. **d,** Evolution of edge state distributions on one face (with vertices q_{12}^2, q_{13}^2 *and* q_{14}^2) of the regular octahedron. Lines/dots indicate numerical/analytical results.

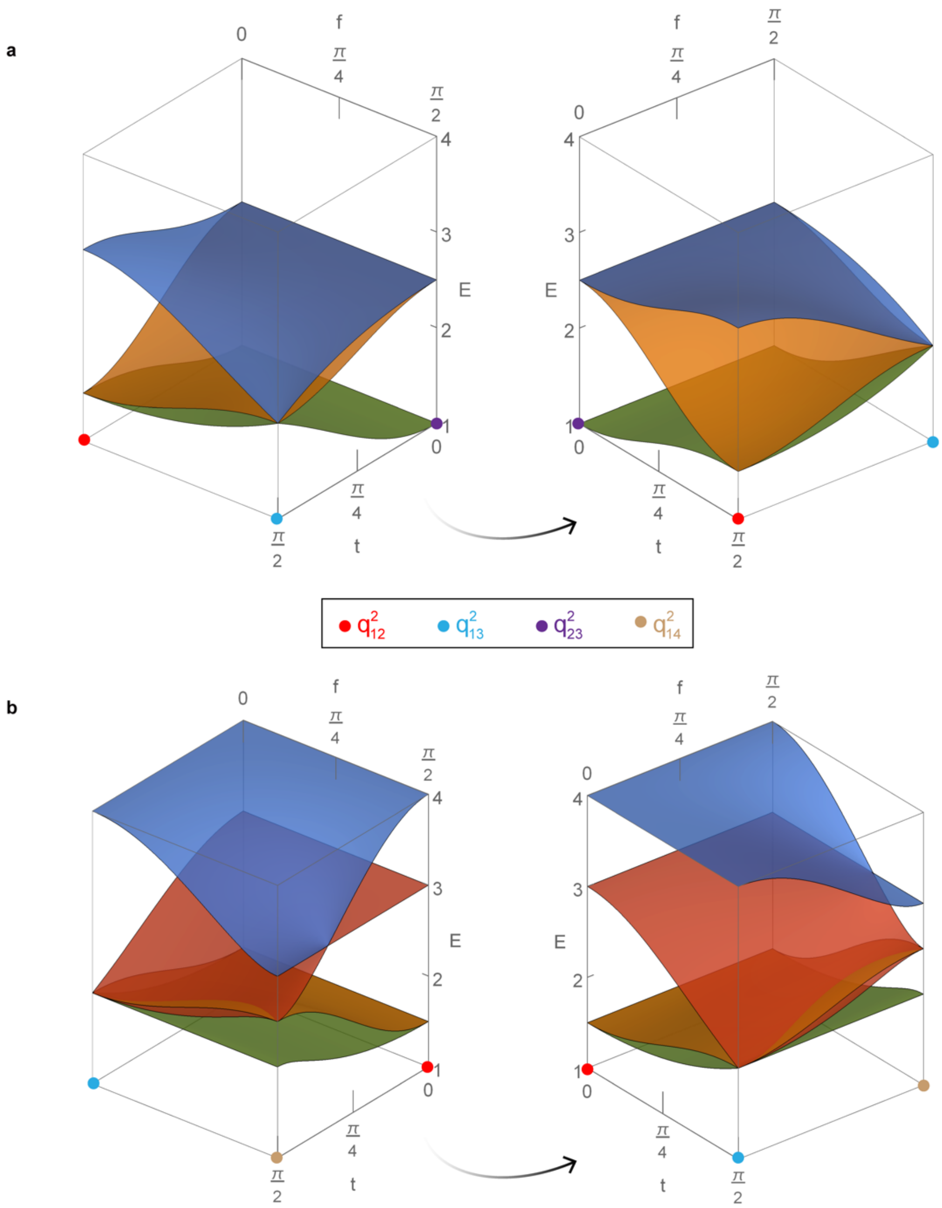

Supplementary Figure 11. Analytical edge state surfaces. a, Edge state surfaces on the triangle face of $(q_{12}^2, q_{13}^2, q_{23}^2)$ involving three bands. b, Edge state surfaces on the triangle face of $(q_{12}^2, q_{13}^2, q_{14}^2)$ involving four bands.

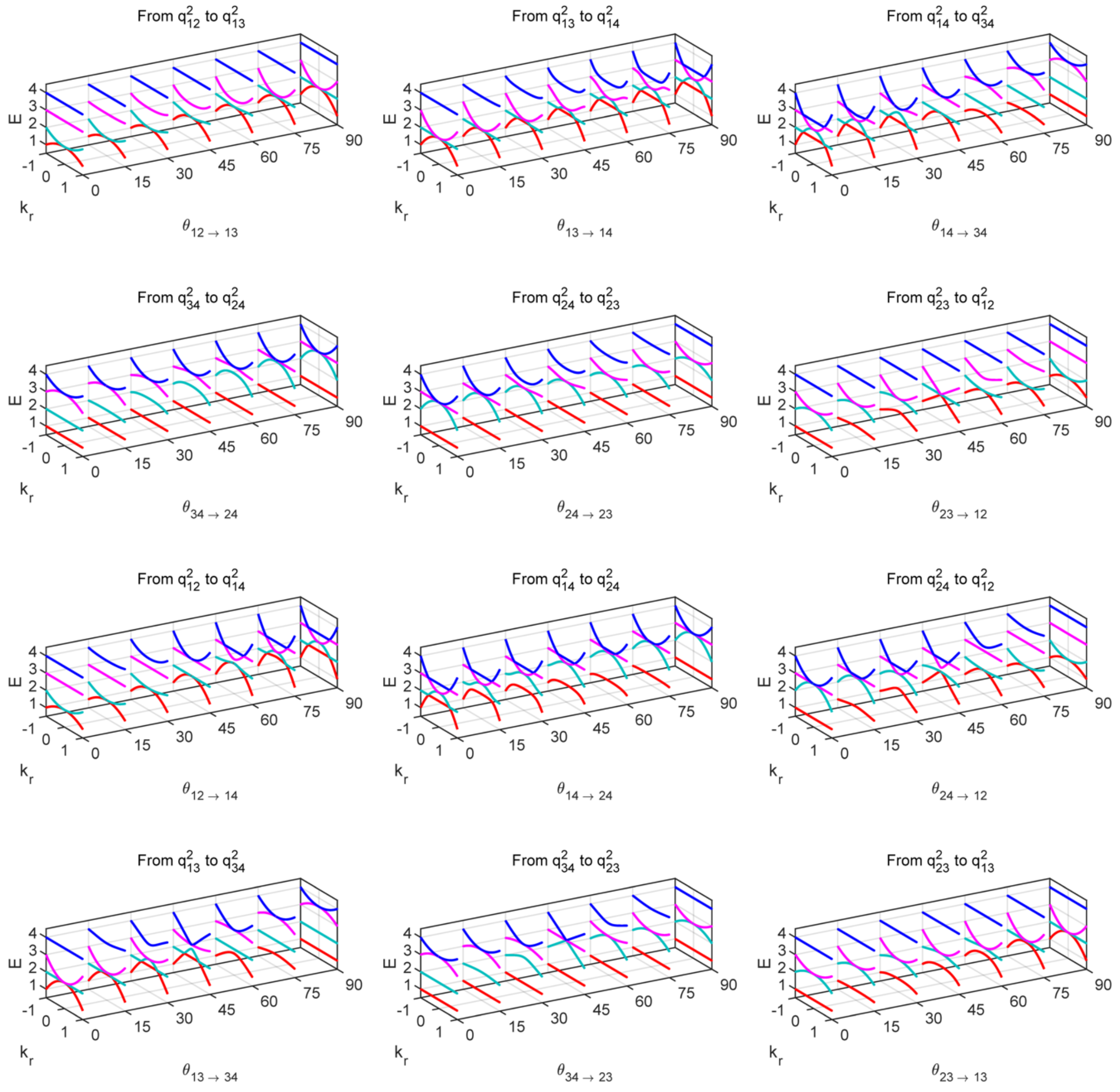

Supplementary Figure 12. The evolution of radial cuts $E(k_r)$ of the extended two-dimensional bands between different factorizations of charge -1. The point degeneracies at $k_r = 0$ can be topologically related to the edge states of the 1D systems shown in Supplementary Figure 10. The other degeneracies ($k_r \neq 0$) are accidental without topological meaning.

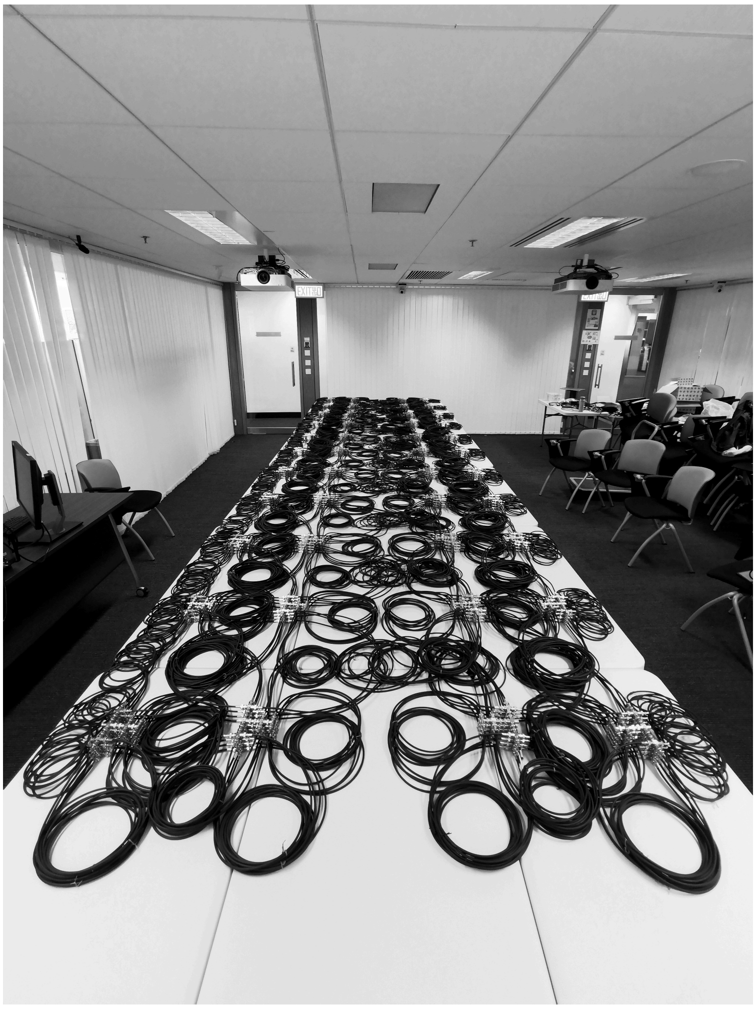

Supplementary Figure 13. Transmission line network constructed for charges $\pm q_{14}$, where around 880 coaxial cables are used.

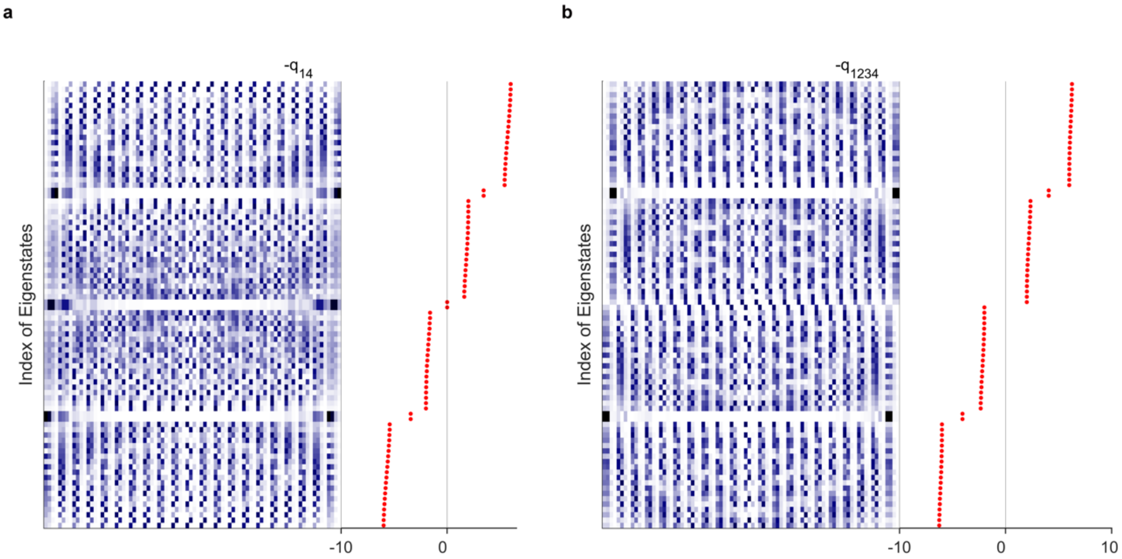

Supplementary Figure 14. Distribution of hard boundary edge states for charges $\pm q_{14}$(a) and $-q_{1234} = -q_{12}q_{34}$(b). Detailed parameters are listed in Supplementary Table 5.

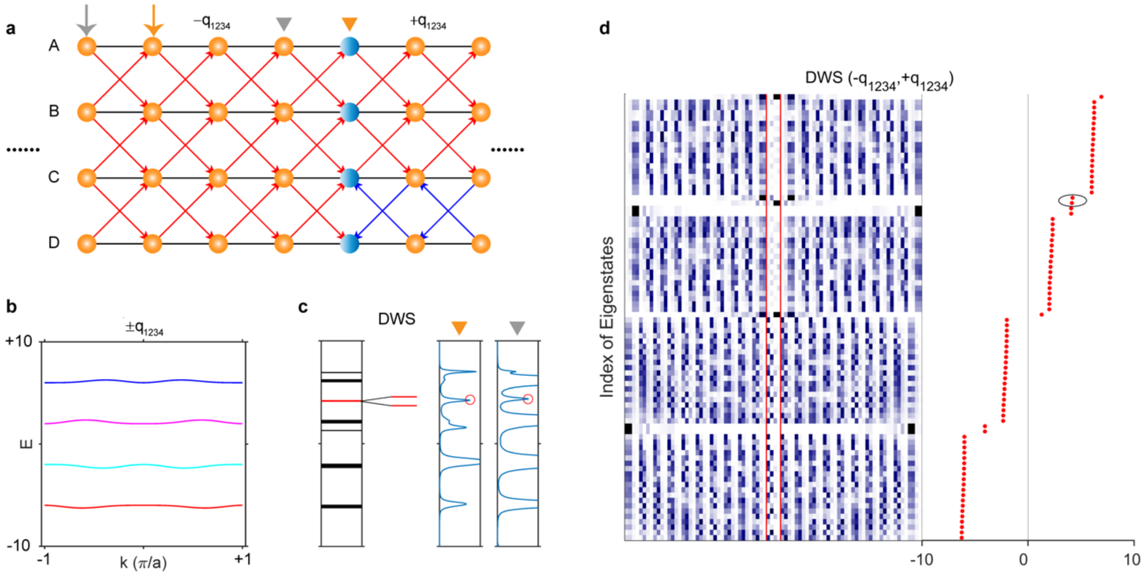

Supplementary Figure 15. Construction of domain-wall (blue spheres in panel a) and distribution of domain-wall states (DWS) indicated by the black ellipse in panel (d). Panels (a) and (c) are copied from the main text (Fig. 4) for comparison purpose. b, Bulk states of $\pm q_{1234}$, they are overlapped. Detailed parameters are listed in Supplementary Table 5 (see the column of $-q_{12}q_{34}$). For charge q_{1234}, we set $w_{CD} = -1$. We say that one domain-wall state locating

in the second bandgap and another one with energy beyond the bulk spectrum are induced by the domain-wall construction, and thus topologically trivial.

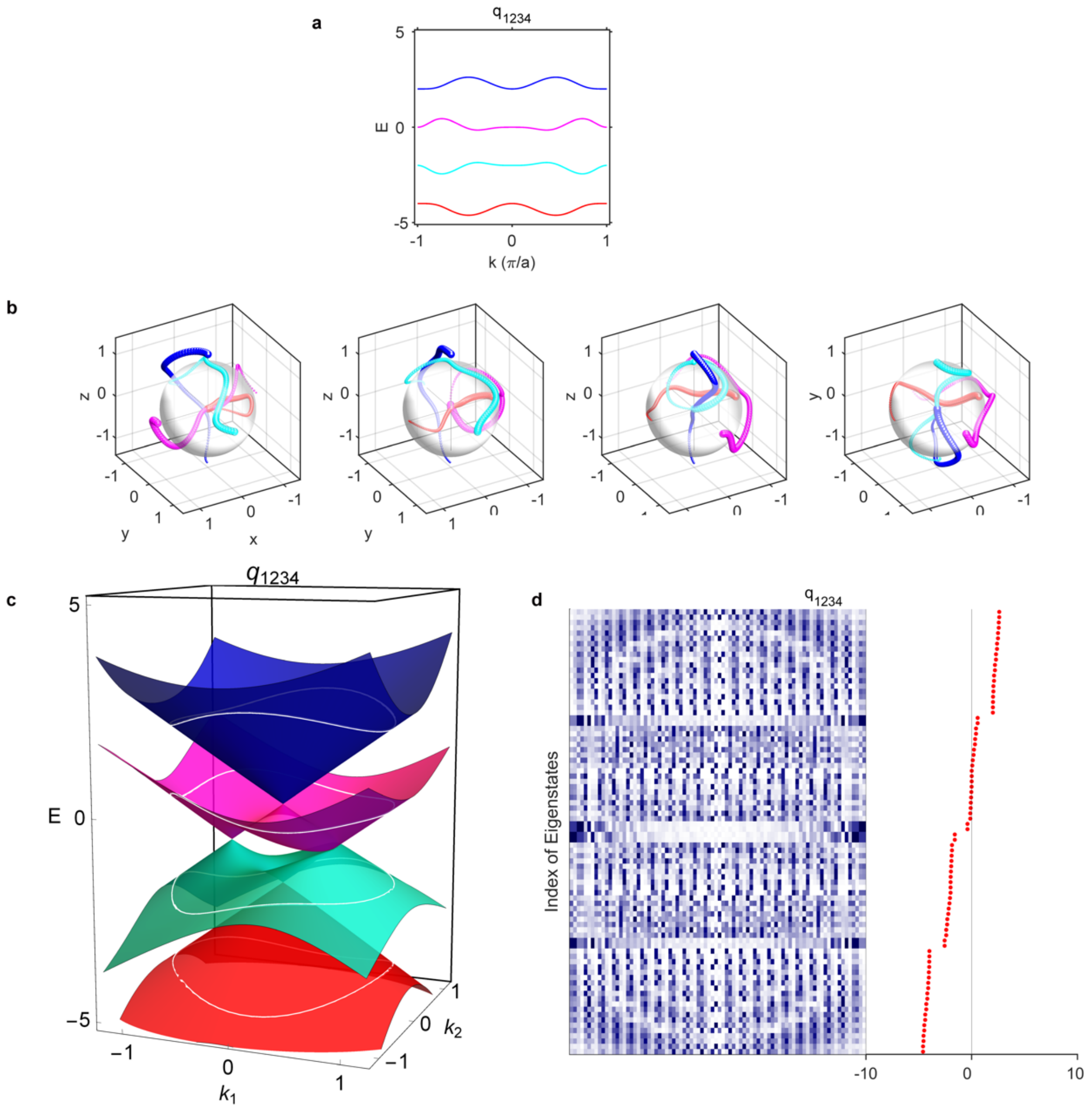

Supplementary Figure 16. The case of $q_{1234} = -q_{13}q_{24}$. a, Bulk states. b, Trajectories of four eigenstates as wavevector runs across the first Brillouin zone ($k = -\pi \rightarrow \pi$). c, The extended energy bands on a 2D plane, where the white circles indicate the corresponding 1D energy bands. d, Distribution of hard boundary edge states. The first, second, third and fourth bands are coloured as red, cyan, magenta and blue, respectively. There is one linear Dirac cone between the first/third and second/fourth bands, and two linear Dirac cones between the second and third bands. Each linear Dirac cone implies one corresponding edge state per edge.

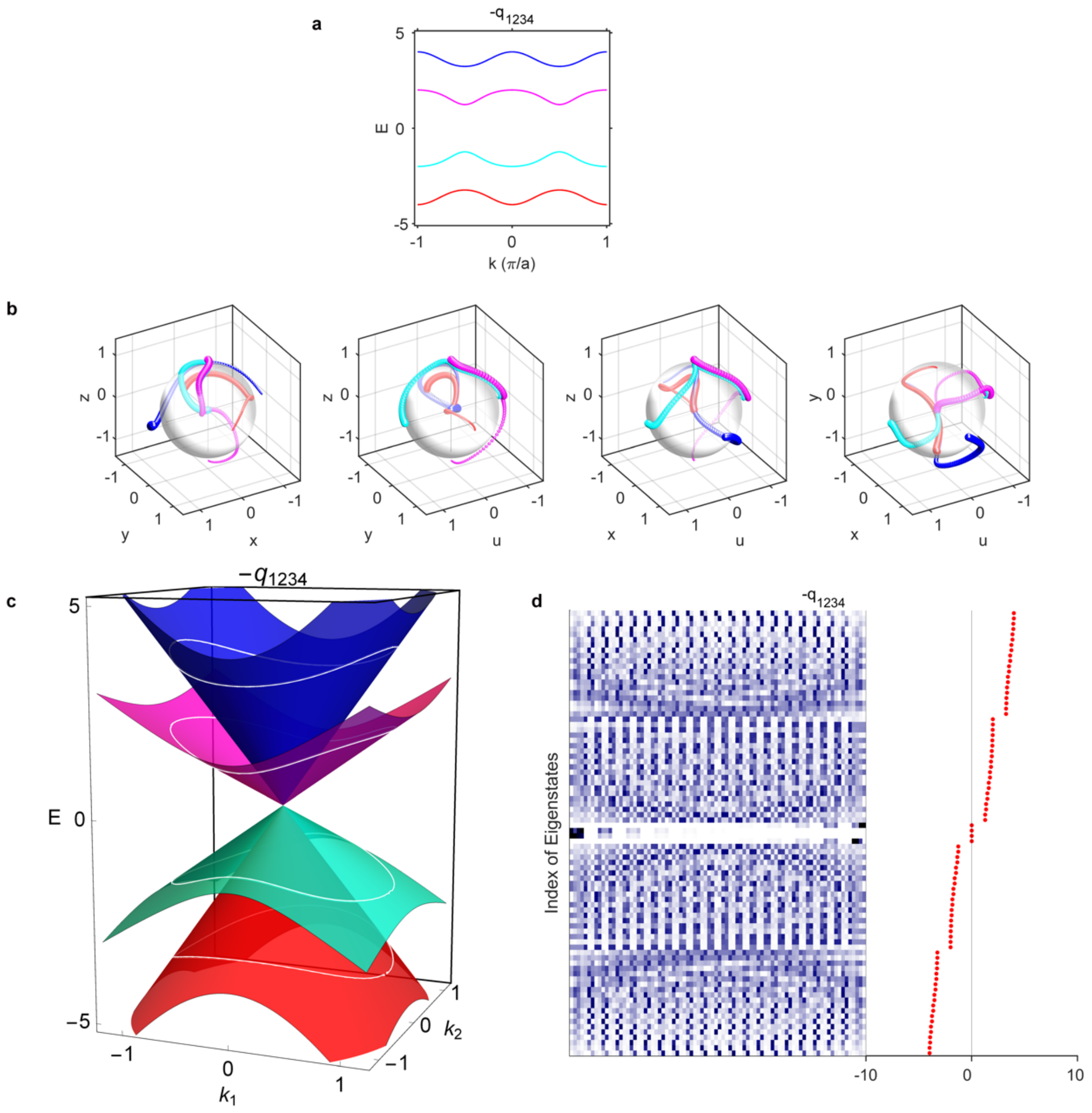

Supplementary Figure 17. The case of $-q_{1234} = -q_{14}q_{23}$. a, Bulk states. b, Trajectories of four eigenstates as wavevector runs across the first Brillouin zone ($k = -\pi \rightarrow \pi$). c, The extended energy bands on a 2D plane, where the white circles indicate the corresponding 1D energy bands. d, Distribution of hard boundary edge states. The first, second, third and fourth bands are coloured as red, cyan, magenta and blue, respectively. There is one four-fold linear Dirac cone between the four bands, which implies two edge states per edge.

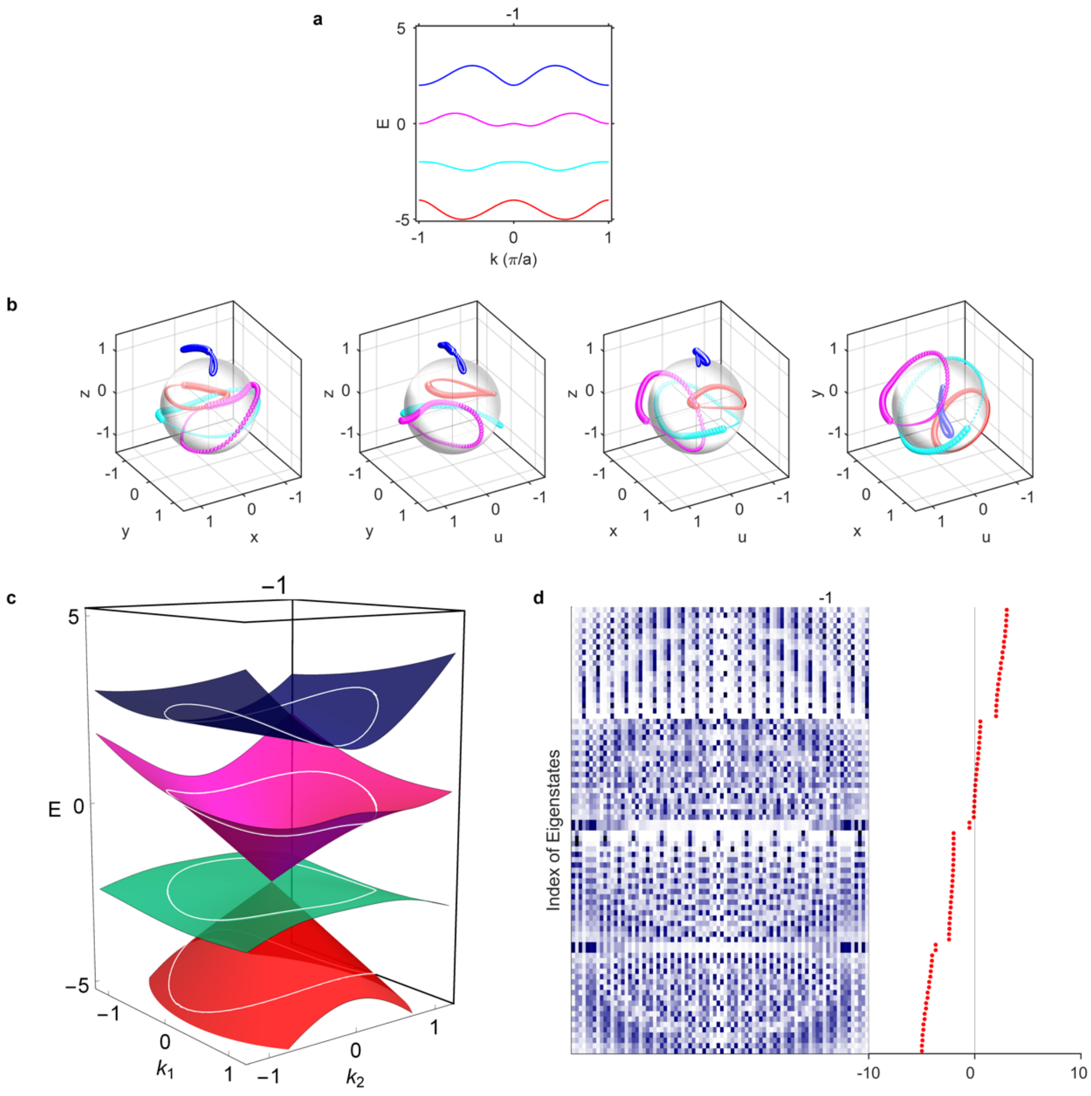

Supplementary Figure 18. The case of charge -1. a, Bulk states. b, Trajectories of four eigenstates as wavevector runs across the first Brillouin zone ($k = -\pi \rightarrow \pi$). c, The extended energy bands on a 2D plane, where the white circles indicate the corresponding 1D energy bands. d, Distribution of hard boundary edge states. The first, second, third and fourth bands are coloured as red, cyan, magenta and blue, respectively. There is one triple linear degeneracy constructed by the lower three bands, which implies that for the first and second bandgaps each supports one edge state per edge, being similar to some cases of charge -1 in three-band models[5].
